# SemiLT: A Multianchor Transfer Learning Method for Cross‐Modality Cell Label Annotation from scRNA‐seq to scATAC‐seq

**DOI:** 10.1002/advs.202507846

**Published:** 2025-09-02

**Authors:** Zhitong Chen, Maoteng Duan, Xiaoying Wang, Bingqiang Liu

**Affiliations:** ^1^ School of Mathematics Shandong University Jinan Shandong 250100 China; ^2^ Shandong National Center for Applied Mathematics Jinan Shandong 250100 China; ^3^ State Key Laboratory of Cryptography and Digital Economy Security Shandong University Jinan Shandong 250100 China

**Keywords:** label transfer, multi‐anchor batch correction, scATAC‐seq, scRNA‐seq, transfer learning

## Abstract

scATAC‐seq enables the detailed exploration of epigenetic variations across various cell clusters, providing complementary insights to scRNA‐seq. However, its extreme sparsity and high dimensionality pose significant challenges for cell type annotation. Transfer learning can extract key features from well‐annotated data to assist in annotating target data, thereby improving annotation accuracy. However, existing transfer learning methods overlook the temporal discrepancies between scRNA‐seq and scATAC‐seq, which exacerbate batch effects between these two modalities. Therefore, SemiLT, a multi‐anchor transfer learning method, is introduced for cell label annotation from scRNA‐seq to scATAC‐seq. Benchmarking across multiple datasets shows that SemiLT outperforms existing tools in both cell type annotation and modality batch correction. Notably, the F1 score for rare cell types improves by an average of 18%. The high‐quality annotation and embedding provided by SemiLT enhance the reliability of downstream analyses. When applied to the human bone marrow hematopoietic dataset, the trajectory transitions of hematopoietic stem cells (HSCs) are accurately reconstructed. Similarly, when applied to human peripheral blood mononuclear cell (PBMC) datasets, the key low‐abundance transcription factor (TF) KLF4 is identified in CD8 effector T cells through label transfer from scRNA‐seq to scATAC‐seq, a result that is difficult to achieve using scRNA‐seq data alone.

## Graphical abstract

A multianchor transfer learning method, SemiLT, enables accurate cell label annotation in sparse scATAC‐seq data by leveraging well‐annotated scRNA‐seq references. By addressing temporal discrepancies between modalities, SemiLT improves rare cell type identification and batch correction. Applications to hematopoietic and immune datasets highlight its potential to uncover biological signals that are difficult to detect using scRNA‐seq alone.

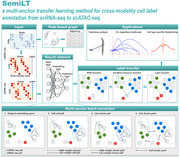

## 1 Introduction

The advent of single‐cell ATAC sequencing (scATAC‐seq) provides researchers with unprecedented opportunities to explore chromatin accessibility at the single‐cell level,^[^ ^1^ ^]^ advancing the identification of key regulatory elements essential for complex diseases.^[^ ^2^ ^]^ However, the extreme sparsity, high dimensionality, lack of reliable cell markers in scATAC‐seq data make it difficult to perform cell type annotation based on scATAC‐seq.^[^ ^3^ ^]^ In contrast, single‐cell RNA sequencing (scRNA‐seq), as the most widely used method in single‐cell omics, is supported by reliable cell markers and has been extensively annotated and classified into different cell atlases.^[^ ^4^, ^5^ ^]^ This inspired us to transfer cell labels from scRNA‐seq to scATAC‐seq, thereby enhancing our understanding of the relationship between chromatin structure and its functionality in various biological processes.

Existing label transfer methods can be classified into two categories based on their strategies of obtaining a joint embedding space for two modalities. The first category of methods first applies unsupervised joint dimensionality reduction or batch effect correction on well‐annotated scRNA‐seq and unannotated scATAC‐seq to construct a joint embedding space, such as Seurat,^[^ ^6^ ^]^ Conos,^[^ ^7^ ^]^ and scMODAL.^[^ ^8^ ^]^ The second category employs a semi‐supervised approach during the embedding process, leveraging cell labels from scRNA‐seq to refine the scATAC‐seq embedding, such as scJoint,^[^ ^9^ ^]^ scNLC,^[^ ^10^ ^]^ NeuCA,^[^ ^11^ ^]^ and scGT.^[^ ^12^ ^]^ Both types of methods share a common feature: they reduce batch effects between the two modalities using a single type of cell‐cell anchors or other alignment approaches, followed by cell label transfer in the embedding space. However, existing tools overlook the temporal discrepancies between the two modalities, where changes in chromatin accessibility precede gene expression,^[^ ^13^ ^]^ and the changes in gene expression can be considered as the integral of the rate of chromatin accessibility changes. This temporal discrepancy does not result from PCR amplification bias, sequencing depth, or other experimental technical factors, but arises from the inherent biological timing differences between the modalities. These temporal discrepancies substantially intensify batch effects between scRNA‐seq and scATAC‐seq. Existing label transfer methods typically rely on a single type of cell–cell anchor, which limits the flexibility in tuning batch correction strength and can lead to over‐correction or under‐correction under temporal mismatch. Moreover, existing methods, such as scJoint, scNLC and scMODAL, rely on KNN classifiers to annotate scATAC‐seq, which tends to bias the classification toward major cell types, often at the expense of rare ones. These factors limit the accuracy of cell type annotation in scATAC‐seq.

To address the existing challenges, we introduced SemiLT, a transfer learning model that integrates semi‐supervised learning and a multi‐anchor batch correction method. SemiLT extracts key features from well‐annotated scRNA‐seq data that help identify and classify cell types and transfers both major and rare cell labels from scRNA‐seq to scATAC‐seq. SemiLT first constructs multiple cell anchors in the embedding space to align scRNA‐seq and scATAC‐seq while preserving cell‐to‐cell similarity within scATAC‐seq. Secondly, SemiLT assigns different weights and functions to the cell anchors to reduce batch effects arising from temporal factors between the two modalities. Finally, SemiLT introduces a Euclidean distance classifier to balance the K‐Nearest Neighbors (KNN) classifier, ensuring the accurate annotation of rare cell types. Benchmarking across seven datasets shows that SemiLT outperforms existing methods in six metrics for evaluating cell clustering and batch correction. Notably, based on the cell‐type annotations provided by SemiLT, the trajectory transitions of hematopoietic stem cells (HSCs) were accurately reconstructed from the scATAC‐seq data in a human bone marrow hematopoiesis dataset, and the key low‐abundance transcription factor (TF) KLF4 was identified in CD8 effector T cells in the PBMC dataset.

## 2 Results

### 2.1 Overview of the SemiLT Framework

**Figure 1:**
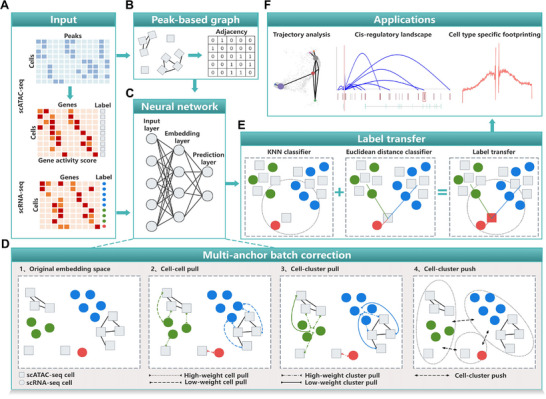
The framework of SemiLT. A) SemiLT uses the scATAC‐seq, GAS, and annotated scRNA‐seq as input. B) SemiLT constructs a cell graph in the peak space for scATAC‐seq. C) SemiLT inputs both scRNA‐seq and scATAC‐seq cells into a fully connected neural network. D) SemiLT aligns scRNA‐seq and scATAC‐seq in the embedding space using a multi‐anchor batch correction method, combining cell/cluster pull (attraction) and cluster push (repulsion) to structure the embedding space. E) Label transfer is performed through the KNN classifier and Euclidean distance classifier. F) SemiLT‐annotated scATAC‐seq can be used for downstream analyses such as trajectory analysis, cell type‐specific cis‐regulatory landscape, and cell type‐specific footprinting.

SemiLT establishes a cross‐modality annotation framework that transfers cell labels from well‐annotated scRNA‐seq to scATAC‐seq using a neural network based on transfer learning. Firstly, it takes three types of input data: scRNA‐seq, scATAC‐seq, and gene activity scores (GAS) derived from scATAC‐seq (**Figure** **1**A). Secondly, it constructs a graph based on the similarity of scATAC‐seq cells in the peak space (**Figure** 1B) and inputs both scRNA‐seq and scATAC‐seq cells into a fully connected neural network (**Figure** 1C). Within the network, scRNA‐seq and scATAC‐seq are aligned using a multi‐anchor batch correction method (**Figure** 1D), which combines cell pull, cluster pull, and cluster push to reduce intra‐type distances (pull) and increase inter‐type separation (push) in the embedding space. Finally, it transfers cell labels from scRNA‐seq to scATAC‐seq by integrating a KNN classifier and a Euclidean distance classifier within the common embedding space (**Figure** 1E). The annotations generated by SemiLT can improve downstream analyses such as trajectory inference, cell type‐specific cis‐regulatory analysis, and cell type‐specific transcription factor footprinting analysis (**Figure** 1F).

### 2.2 Benchmarking SemiLT's Performance with Paired Datasets

**Figure 2:**
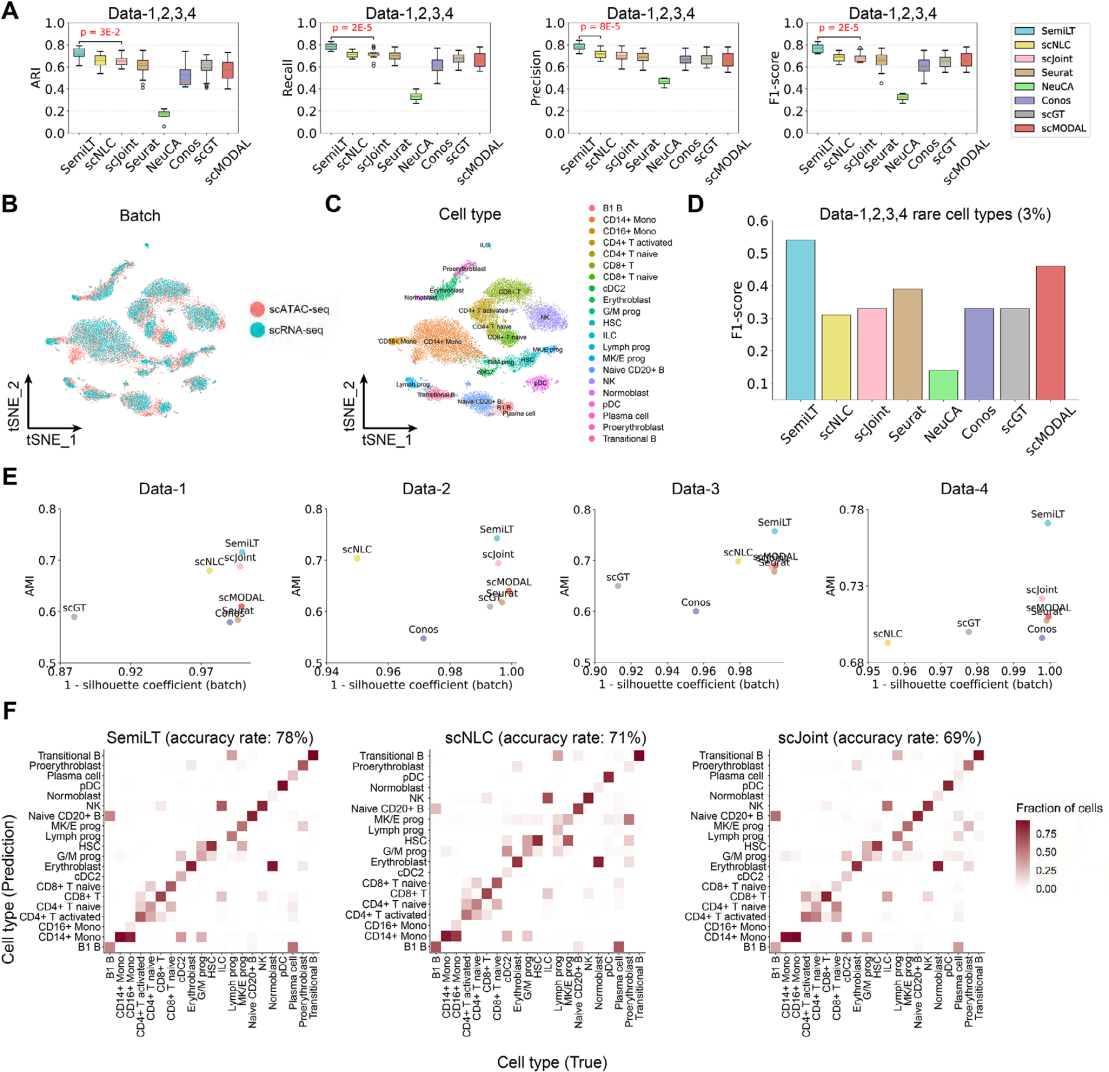
Benchmarking SemiLT's performance with paired datasets. A) ARI, Recall, Precision and F1 score of SemiLT and other methods. B) tSNE visualization of SemiLT integrated data generated from scRNA‐seq and scATAC‐seq colored by technology. C) tSNE visualization of SemiLT integrated data generated from scRNA‐seq and scATAC‐seq colored by true cell labels. D) F1 score for the prediction of rare cell types (3%) across Data‐1,2,3,4. E) AMI and modality silhouette coefficients of SemiLT and other methods across Data‐1,2,3,4. F) Predicted cell types and their fractions of agreement with the original cell types for SemiLT, scNLC and scJoint.

To evaluate SemiLT's performance in annotating scATAC‐seq data with paired datasets, we applied it to four paired scRNA‐seq and scATAC‐seq datasets (Data‐1, 2, 3, 4) from human bone marrow mononuclear cells.^[^ ^14^ ^]^ We compared SemiLT against scNLC, scJoint, Seurat, NeuCA, Conos, scGT and scMODAL evaluating five parameter configurations for each dataset, including the default settings and parameter variations (Note S1, Supporting Information). Across all four paired datasets, SemiLT outperformed all other tools in ARI, Recall, Precision, and F1 score (**Figure** **2**A). To assess whether the observed performance differences were statistically significant, we performed the Wilcoxon rank‐sum test on ARI, recall, precision, and F1 score across datasets. The results confirmed that the improvements achieved by SemiLT were statistically significant (*P* < 0.05) (**Figure** 2A). Furthermore, tSNE plots indicate SemiLT's effective batch effect removal, demonstrating the successful integration of scRNA‐seq and scATAC‐seq data (**Figure** 2B) (Figure S1, Supporting Information). We also present tSNE plots for Data‐1 to Data‐4, colored by cell types, showing that SemiLT preserves biological differences among various cell types (**Figure** 2C) (Figure S2, Supporting Information).

To demonstrate that the embeddings generated by SemiLT enhance the identification of rare cell types, we selected all rare cell types from Data‐1 to Data‐4. Specifically, we defined rare cell types using four thresholds based on their proportions: less than 10%, 5%, 3%, and 1%, respectively.^[^ ^15^ ^]^ The average F1 score across these rare cell types demonstrates that SemiLT achieved the highest performance in identifying rare cell types (**Figure** 2D) (Figure S3, Supporting Information). Additionally, SemiLT achieved a higher AMI and lower modality silhouette coefficients compared to all other methods, further confirming the superiority of its embeddings (**Figure** 2E) (Figure S4, Supporting Information). In conclusion, SemiLT consistently outperformed other methods in predicting both major and rare cell types across all four datasets (**Figure** 2F) (Figures S5–S8, Supporting Information).

### 2.3 Benchmarking SemiLT's Performance with Unpaired Datasets

To demonstrate the performance of SemiLT in annotating scATAC‐seq data using unpaired datasets, we applied it to three independent datasets: 1) the mouse spleen dataset (Data‐5), where scRNA‐seq and scATAC‐seq data were processed using UniPort;^[^ ^16^ ^]^ 2) the mouse cell atlas subset (Data‐6), consisting of scRNA‐seq data from the Tabula Muris atlas^[^ ^4^ ^]^ and scATAC‐seq data from the Cusanovich atlas;^[^ ^17^ ^]^ and 3) the CITE‐seq and ASAP‐seq dataset (Data‐7) from a T cell stimulation experiment conducted by Mimitou et al.^[^ ^18^ ^]^

**Figure 3:**
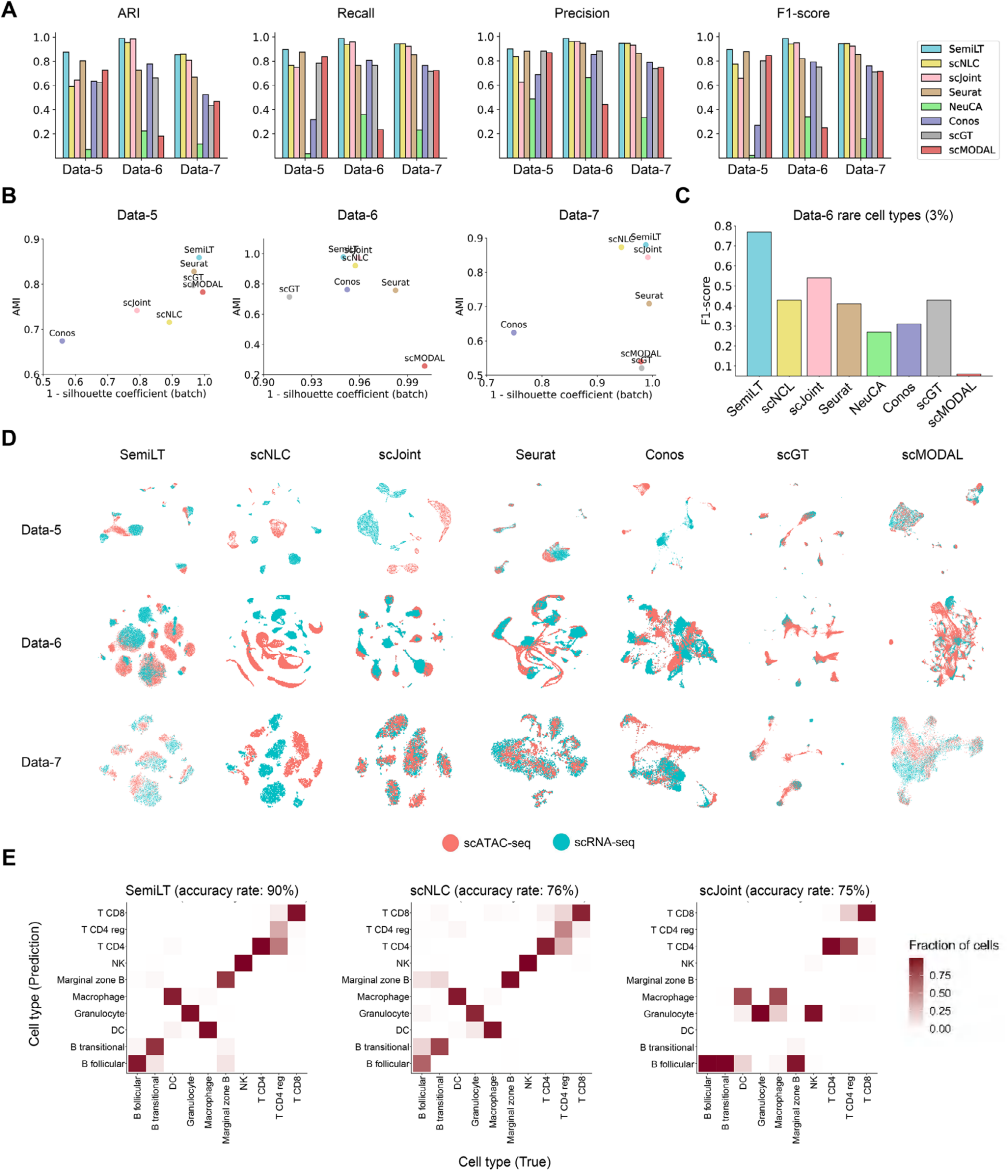
Benchmarking SemiLT's performance with unpaired datasets. A) ARI, Recall, Precision and F1‐score of SemiLT and other methods across Data‐5,6,7. B) AMI and modality silhouette coefficients of SemiLT and other methods across Data‐5,6,7. C) F1 score for the prediction of rare cell types in Data‐6. D) tSNE visualization of SemiLT integrated Data‐5,6,7 generated from scRNA‐seq and scATAC‐seq colored by technology. E) Predicted cell types and their fractions of agreement with the original cell types for SemiLT, scNLC and scJoint.

Across the three unpaired datasets, SemiLT consistently outperformed other methods in terms of ARI, Recall, Precision, and F1 score (**Figure** **3**A). In addition, quantitative evaluation metrics demonstrate that the embeddings generated by SemiLT enable accurate prediction of scATAC‐seq cells while effectively removing batch effects between modalities (**Figure** 3B) (Figure S9, Supporting Information). To assess SemiLT's prediction accuracy for rare cell types, we selected all rare cell types from Data‐6 using the same criteria as before. The average F1 score for these rare cell types demonstrates that SemiLT consistently outperformed other tools in identifying rare cell types, achieving the highest average F1 score (**Figure** 3C) (Figure S10, Supporting Information). Furthermore, tSNE plots highlight SemiLT's ability to remove batch effects (**Figure** 3D), illustrating the integration of scRNA‐seq and scATAC‐seq data while preserving biological differences among various cell types (Figure S11, Supporting Information).

In conclusion, SemiLT demonstrated the highest prediction accuracy for both major and rare cell types across the three unpaired datasets (**Figure** 3E) (Figures S12–S14, Supporting Information).

### 2.4 Identification of Hematopoietic Hierarchy and the Differences in Genomic Regulation

To investigate whether the embeddings generated by SemiLT can facilitate the inference of cellular trajectory transitions, we applied it to an unpaired human developmental hematopoiesis dataset (Data‐8) to explore the trajectory transitions of HSCs. The scATAC‐seq data, which lack ground‐truth labels, were obtained from Maria Ranzoni,^[^ ^19^ ^]^ while the well‐annotated scRNA‐seq data were obtained from Setty et al.^[^ ^20^ ^]^

**Figure 4:**
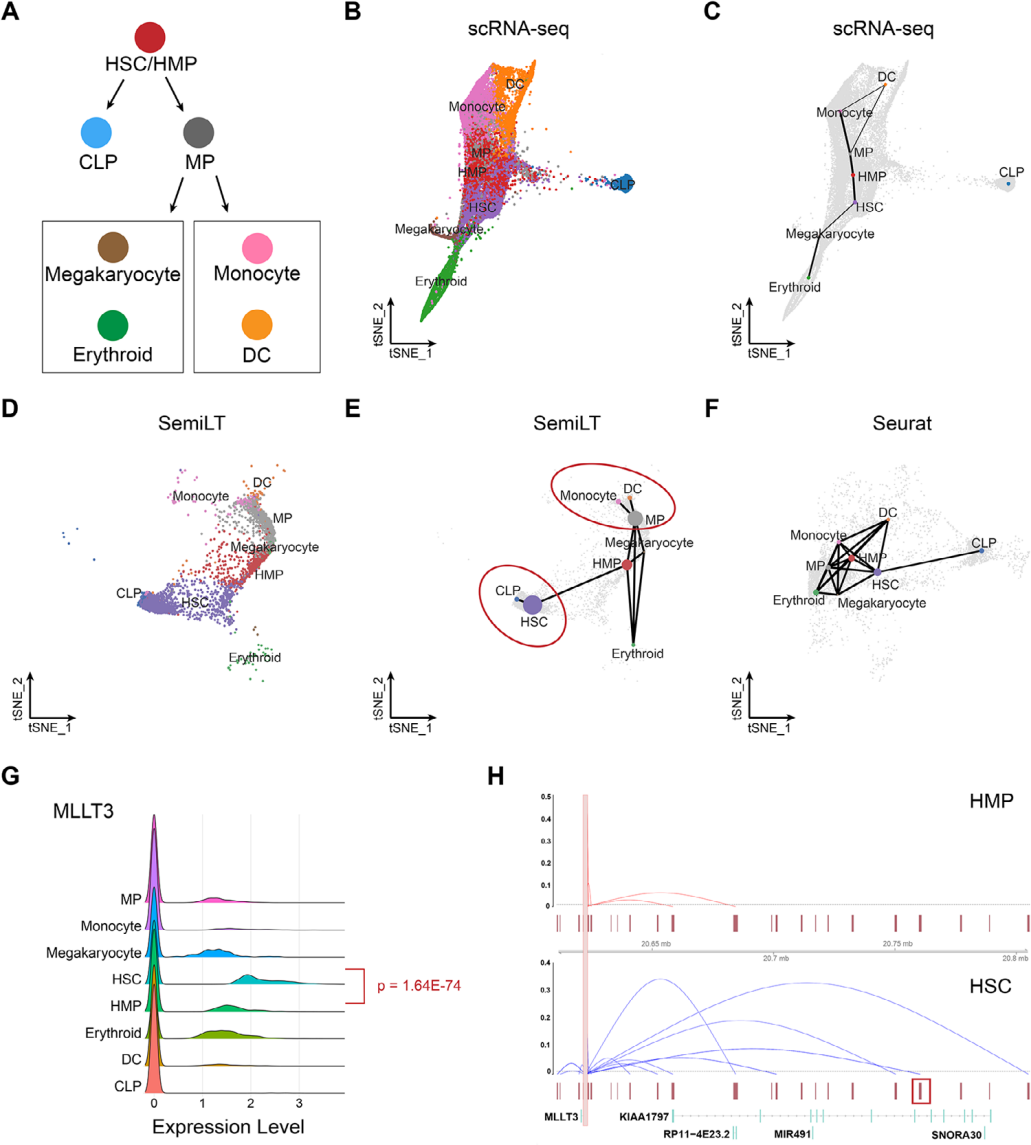
Trajectory transitions and cis‐regulatory differences in HSC. A) Ground truth for the trajectory transition of HSC. B) tSNE visualization of scRNA‐seq, colored by true cell types. C) Cell trajectory inferred from the embedding of scRNA‐seq. D) tSNE visualization of SemiLT‐annotated scATAC‐seq, colored by predicted cell types. E) Cell trajectory inferred from the embedding of SemiLT. F) Cell trajectory inferred from the embedding of Seurat. G) Ridge plots of the MLLT3 gene in scRNA‐seq. H) A summary of the Cicero co‐accessibility links between the MLLT3 promoter and distal sites in the surrounding region from scATAC‐seq annotated by SemiLT.

HSCs follow two distinct differentiation trajectories: one differentiates into common lymphoid progenitors (CLPs), while the other first differentiates into myeloid progenitors (MPs), which further branch into two pathways—one leading to erythrocytes and megakaryocytes, and the other to dendritic cells (DCs) and monocytes^[^ ^21^ ^]^ (**Figure** **4**A). However, when we performing PAGA^[^ ^22^ ^]^ trajectory inference based solely on scRNA‐seq (**Figure** 4B), we failed to reconstruct the differentiation trajectory from HSCs to CLPs (**Figure** 4C). In contrast, the cell embeddings generated by SemiLT integrate both transcriptomic and chromatin accessibility information (**Figure** 4D). When performing PAGA trajectory inference, it not only captures the differentiation trajectory from HSCs to CLPs but also accurately captures the two differentiation branches of MP cells (**Figure** 4E). When using embeddings generated by other methods, such as Seurat, can also reconstruct the differentiation trajectory from HSCs to CLPs, but they tend to introduce more noisy edges, such as an incorrectly edge between DC and Monocyte, which impair the accuracy and biological interpretability of the inferred trajectory (**Figure** 4F).

In addition, the scATAC‐seq annotations generated by SemiLT not only facilitate the inference of HSC trajectory transitions but also enable joint analysis with scRNA‐seq to reveal genome cis‐regulatory differences across different cell types. We performed differential expression analysis between HSCs and hematopoietic multipotent progenitors (HMPs) based on scRNA‐seq using the Wilcoxon rank‐sum test, and found that the gene *MLLT3* is significantly upregulated in HSCs (**Figure** 4G). To investigate the underlying cause of this difference, we performed Cicero^[^ ^23^ ^]^ cis‐regulatory analysis based on the scATAC‐seq annotations from SemiLT. Our analysis revealed that the distal elements linked to the promoter region of the *MLLT3* differ between HSCs and HMPs (**Figure** 4H). We further annotated all distal elements using the scEnhancer^[^ ^24^ ^]^ database and identified a peak region at Chr9: 20759862‐20760953 (highlighted in the red box at the bottom right of **Figure** 4H), which precisely corresponds to the *MLLT3* enhancer. The interaction between this enhancer and the promoter facilitates the recruitment of RNA polymerase, thereby enhancing the expression of *MLLT3*. This regulatory mechanism may contribute to the differential expression of *MLLT3* between HSCs and HMPs.

### 2.5 Identification of KLF4 as a Cryptic Regulator in CD8^+^ T Cell Effector Function

**Figure 5:**
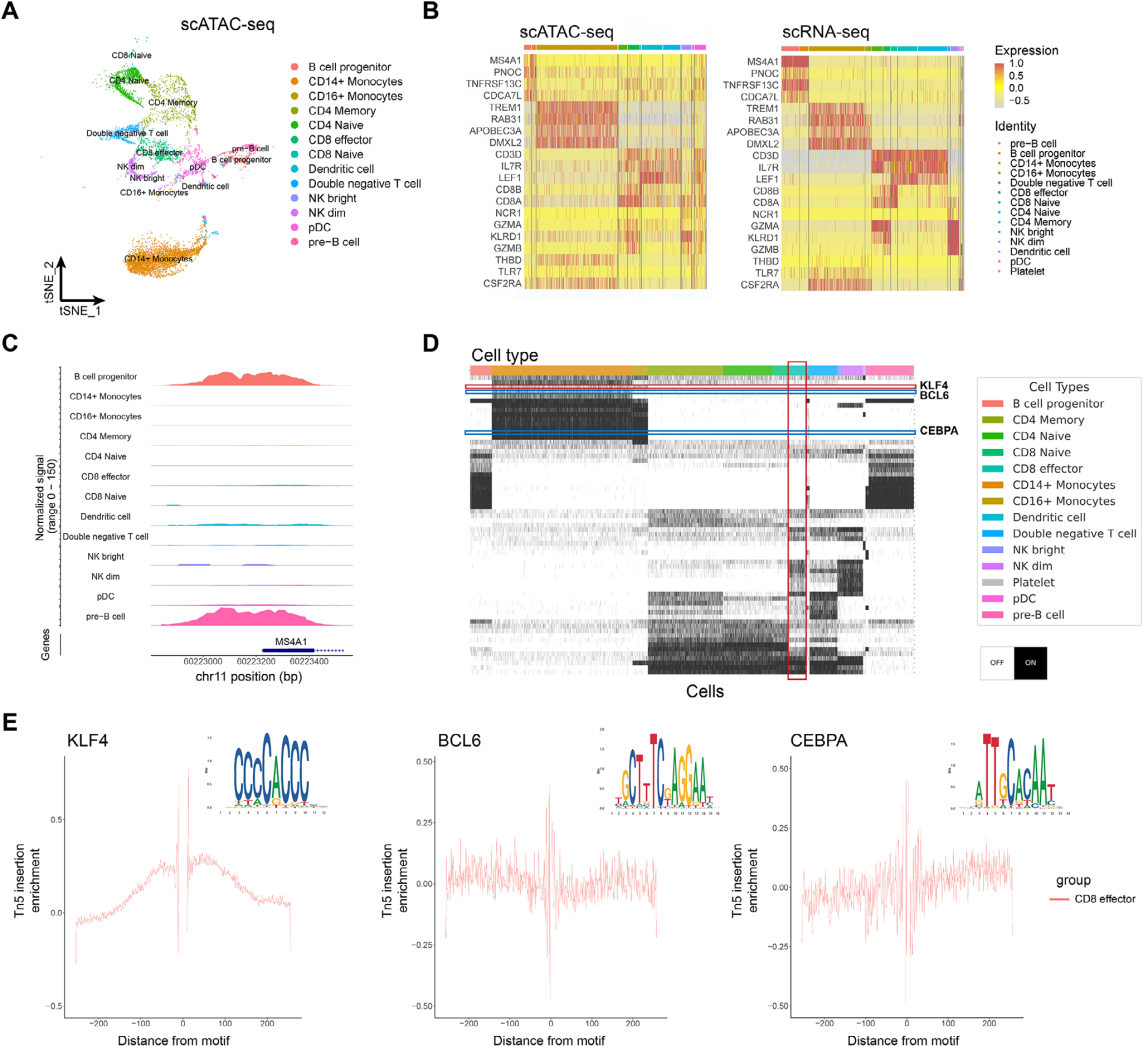
TF regulons of CD8 effector T cells from PBMC. A) tSNE visualization of SemiLT‐annotated scATAC‐seq data, colored by predicted cell types. B) Heatmap of scores of genes and GAS, calculated from cells aggregated by true cell labels in scRNA‐seq (right), predicted cell types by SemiLT (left). C) Plot of Tn5 insertion frequency over the promoter region of MS4A1. D) The scores of TF regulons in cell clusters, calculated by SCENIC. E) TF footprinting analysis for KLF4, BCL6 and CEBPA.

We applied SemiLT to Data‐9,^[^ ^25^ ^]^ an unpaired PBMC dataset in which scATAC‐seq data lack ground‐truth labels, and transferred cell labels from scRNA‐seq to scATAC‐seq. SemiLT annotated cells in the scATAC‐seq into 13 cell types (**Figure** **5**A). We observed that the GAS of marker genes in scATAC‐seq closely resemble the gene expression levels of the corresponding cell types in scRNA‐seq (**Figure** 5B), confirming the accuracy of SemiLT's cell annotations. Furthermore, chromatin accessibility at the promoter regions of marker genes exhibits distinct patterns across different cell types (**Figure** 5C) (Figure S15, Supporting Information).

To investigate the regulatory role of CD8 effector T cells in immune modulation, we applied SCENIC,^[^ ^26^ ^]^ a scRNA‐seq‐based method, to identify key TF regulons within CD8 effector T cells. However, we found that some TF regulons, such as *KLF4*, *BCL6*, and *CEBPA*, exhibited low activity in CD8 effector T cells, with fewer than 5% of cells exhibiting a non‐zero regulon activity score, and were thus considered low‐abundance TF regulons (**Figure** 5D). This likely reflects the low overall expression of their downstream target genes, making it difficult for scRNA‐seq‐based methods to detect their regulatory activity. In contrast, scATAC‐seq can directly reveal TF binding activity through chromatin accessibility, even when regulon expression is minimal. To validate these low‐abundance transcripts, we performed TF footprint analysis on the SemiLT‐annotated scATAC‐seq dataset using Signac.^[^ ^25^ ^]^ Our results revealed a distinct TF footprint for *KLF4* (**Figure** 5E), characterized by motif‐bound regions devoid of Tn5 transposase insertions, while numerous Tn5 insertions were enriched in the surrounding regions. This suggests that *KLF4* may bind to motifs in CD8 effector T cells and regulate the transcription of target genes, highlighting the limitation of scRNA‐seq in capturing the functional significance of low‐abundance transcripts.^[^ ^19^ ^]^ This finding is further supported by the study of Nah et al.^[^ ^27^ ^]^ In contrast, low‐abundance transcripts such as *BCL6* and *CEBPA* (**Figure** 5E) did not exhibit distinct TF footprints, suggesting that their regulatory roles in CD8 effector T cells may be limited or less pronounced.

## 3 Discussion

We present SemiLT, a semi‐supervised transfer learning method designed to transfer cell labels from scRNA‐seq to scATAC‐seq. SemiLT constructs a joint embedding space integrating scRNA‐seq, scATAC‐seq, and GAS, and sequentially aligns cells using a multi‐anchor batch correction strategy. This approach offers flexibility in tuning batch correction strength and effectively mitigates both over‐correction and under‐correction, particularly under temporal mismatches. Ablation studies were conducted to assess the contribution of individual components: the impact of the loss function on label transfer performance was evaluated (Figure S16, Supporting Information), and the effectiveness of the Euclidean distance‐based classifier in accurately annotating rare cell types was confirmed (Figure S17, Supporting Information).

Experiments across various datasets demonstrate the superior performance of SemiLT, surpassing existing tools in annotating both major and rare cell types. Six evaluation metrics, encompassing both cell clustering and batch correction, further support this conclusion. In the human developmental hematopoiesis dataset, SemiLT's cell embeddings uniquely reconstruct the trajectory transition from HSC to CLP. Additionally, joint analysis of well‐annotated scRNA‐seq and SemiLT‐annotated scATAC‐seq revealed genomic regulatory differences in the *MLLT3* gene across cell types. In the PBMC dataset, SemiLT‐annotated scATAC‐seq identified the key TF regulon *KLF4* in CD8+ effector T cells, which was undetectable in scRNA‐seq alone.

Despite its advantages, SemiLT has areas for further improvement. On one hand, SemiLT relies on a predefined GAS from external tools to bridge the heterogeneity gap between scRNA‐seq and scATAC‐seq. However, the quality of GAS depends on the accuracy of these external tools, which we aim to enhance in future work. On the other hand, while incorporating scRNA‐seq, GAS, and scATAC‐seq as inputs maximizes the preservation of individual cell information, it also increases the model's computational complexity. This results in extended training times, particularly for large datasets, making computational efficiency optimization a key focus for future improvements.

## 4 Conclusions 

In conclusion, we introduce SemiLT, a semi‐supervised transfer learning method for transferring cell labels from scRNA‐seq to scATAC‐seq. By integrating supervised learning on scRNA‐seq with a multi‐anchor batch correction method, SemiLT outperforms existing state‐of‐the‐art methods in annotating both major and rare cell types. Moreover, SemiLT holds great potential for advancing the joint analysis of gene expression and chromatin accessibility, providing new insights into gene regulatory mechanisms.

## 5 Experimental Section

### Preliminary

SemiLT takes three types of input data, one is the annotated scRNA‐seq, which includes a gene expression matrix *X^r^* with *n*_1_ cells and a corresponding cell label vector *Y*. The other is the unannotated scATAC‐seq, which includes a low‐dimensional representation matrix *X^p^* with *m* features, obtained after PCA dimensionality reduction, and a GAS matrix *X^a^* with *n*_2_ cells, calculated from the scATAC‐seq (Note S2, Supporting Information). Assume suitable intersections have been taken so that *X^r^* and *X^a^* have the same set of genes.

The matrices are represented as follows:

(1) $$X^{r}={\left[{x^{r}}_{1},\text{…},{x^{r}}_{n_{1}}\right]}^{T},\;{x^{r}}_{i}\in R^{g\times 1}$$

(2) $$X^{a}={\left[{x^{a}}_{1},\text{…},{x^{a}}_{n_{2}}\right]}^{T},{x^{a}}_{i}\in R^{g\times 1}$$

(3) $$X^{p}={\left[{x^{p}}_{1},\text{…},{x^{p}}_{n_{2}}\right]}^{T},{x^{p}}_{i}\in R^{m\times 1}$$

(4) $$Y=\left[y_{1},\text{…},y_{n_{1}}\right],y_{i}\in \left\{C_{1},C_{2},\text{…},C_{K}\right\}$$

where *g* is the number of genes, *K* is the number of cell clusters in *X^r^*.

At each training step, a minibatch *B* is constructed by sampling equal‐sized subsets of cells from both datasets, that is $B=B^{r}\cup B^{a}$, where $B^{r}={\left\{{x^{r}}_{i}\right\}}_{i=1}^{n}$ represents *n* annotated scRNA‐seq cells, $B^{a}={\left\{{x^{a}}_{j}\right\}}_{j=1}^{n}$ represents *n* unannotated scATAC‐seq cells. In SemiLT, the neural network is parameterized by a set of weights and biases, collectively denoted as θ. Let ${\left(B_{f_{1}}\right)}_{i,\cdot }=f_{1}\left(x_{i},\theta \right)$ $\in R^{d}$ be the output of the embedding layer when the input $x_{i}\in$ *B* has gone through a transformation of *f*_1_ parametrized by θ, where *d* is the embedding dimensionality and satisfies *d* ≪ *g*. Similarly, the prediction layer *f*_2_ uses the embedding feature as input and outputs *K*‐class probability score ${\left(B_{f_{2}}\right)}_{i,\cdot }=f_{2}\left(f_{1}\left(x_{i},\theta \right)\right)\in R^{K}$. Where *f*_2_ applies the softmax transformation to the output. Finally, the predicted class $\hat{y}$ is defined as:

(5) $$\hat{y}=\arg\max_{j}{\left(B_{f_{2}}\right)}_{i,j}$$

where ${\left(B_{f_{2}}\right)}_{i,j}$ denotes the value of the *j*‐th dimension of the output of the *i*‐th cell after passing through the prediction layer *f*_2_ of the neural network.

### Dimensionality Reduction Loss

For scRNA‐seq, SemiLT employs the loss function *L*_rna_ to improve the separation between cell clusters in the embedding space (the first and second terms), enhance intra‐cluster similarity (the third term), establish an orthogonal embedding space (the fourth term), and anchor the mean of all coordinates near zero (the final term) to ensure model identifiability.

(6) $$\begin{array}{ccc} & & L_{rna}\left(B_{f1}^{r}\right)=-w_{1}\times \frac{\sum_{C_{i}\neq C_{j}},D_{C_{i},C_{j}}}{{\left|C_{B^{r}}\right|}^{2}}+\left(1-w_{1}\right)\\ & & \;\times {\left(\frac{\sum_{j=1}^{d}\sigma {\left(B_{f_{1}}^{r}\right)}_{\cdot ,j}}{d}\right)}^{-1}+\left(\frac{\sum_{i=1}^{\left|C_{B^{r}}\right|}\sum_{j=1}^{d}\sigma {\left(B_{f_{1}}^{r,C_{i}}\right)}_{\cdot ,j}}{\left|C_{B^{r}}\right|\times d}\right)\\ & & \;+w_{2}\times \frac{\sum_{d_{i}\neq d_{j}}{\left|Cov\left(B_{f_{1}}^{r}\right)\right|}_{d_{i},d_{j}}}{d^{2}}+w_{3}\times \frac{\sum_{i=1}^{n}\sum_{j=1}^{d}{\left|B_{f_{1}}^{r}\right|}_{i,j}}{n\times d} \end{array}$$

where $|C_{B^{r}}|$ denotes the number of cell clusters contained in *B^r^*.$D_{C_{i},C_{j}}$ denotes the average pairwise distance between all cells in cluster *C_i_* and all cells in cluster *C_j_*. $\sigma {\left(B_{f1}^{r}\right)}_{\cdot ,j}$ denotes the standard deviation of the values of the feature *j* across different cells in the $B_{f_{1}}^{r}$. $B_{f_{1}}^{r,C_{i}}$ denote the submatrix formed by the cells belonging to the *i*‐th cluster *C_i_*. $Cov\left(B_{f_{1}}^{r}\right)\in R^{d\times d}$ denotes the feature‐wise covariance matrix computed from $B_{f_{1}}^{r}$. *w*_1_,*w*_2_ and *w*_3_ denote the weights of different loss terms. $B_{f_{1}}^{r}\in R^{n\times d}$ denotes the embedding matrix of scRNA‐seq cells in the batch.

For scATAC‐seq, we aim to apply the same loss function as scRNA‐seq.

(7) $$\begin{array}{ccc} L_{\mathrm{gas}} & & \left(B_{f_{1}}^{a}\right)=\left(1-w_{1}\right)\times {\left(\frac{\sum_{j=1}^{d}\sigma {\left(B_{f_{1}}^{a}\right)}_{\cdot ,j}}{d}\right)}^{-1}\\ & & +\left(\frac{\sum_{i=1}^{n}\sum_{j=1}^{d}\sigma {\left(B_{f_{1}}^{a,N\left(i\right)}\right)}_{\cdot ,j}}{n\times d}\right)+w_{2}\times \frac{\sum_{d_{i}\neq d_{j}}{\left|\mathrm{Cov}\left(B_{f_{1}}^{a}\right)\right|}_{d_{i},d_{j}}}{d^{2}}\\ & & +w_{3}\times \frac{\sum_{i=1}^{n}\sum_{j=1}^{d}{\left|B_{f_{1}}^{a}\right|}_{i,j}}{n\times d} \end{array}$$

where N(*i*) denotes the set consisting of cell *i* and its neighbors in the peak space. $B_{f_{1}}^{a}$ denotes the embedding matrix of scATAC‐seq cells in the batch.

### Multi‐Anchor Batch Correction Loss

SemiLT utilizes mutual nearest neighbors (MNN) to construct anchors between scRNA‐seq and scATAC‐seq cells. Cell pairs that directly satisfy the MNN relationship are defined as high‐weight anchors. For scATAC‐seq cells that do not have high‐weight anchors themselves but have neighboring cells in *X^p^* with high‐weight anchors, SemiLT constructs low‐weight anchors under two conditions: 1) their neighboring scATAC‐seq cells are connected to scRNA‐seq cells via high‐weight anchors; and 2) the scATAC‐seq cell is closer (in the embedding space) to the centroid of the scRNA‐seq cell cluster matched by its neighbors than to any other scRNA‐seq cluster centroid. When these conditions are met, SemiLT constructs low‐weight anchors, linking each scATAC‐seq cell to the corresponding scRNA‐seq cell.

SemiLT uses the loss function *L_high_* to enhance the similarity between cell–cell and cell–cluster pairs within high‐weight anchors:

(8) $$\begin{array}{c} \begin{array}{c} L_{high}\left(B_{f_{1}}^{a},B_{f_{1}}^{r}\right)=\frac{\sum_{i\in A_{high}}\sum_{j=1}^{d}\left|{\left(B_{f_{1}}^{a}\right)}_{i,j}-{\left(B_{f_{1}}^{r}\right)}_{A_{high}\left(i\right),j}\right|}{n\times d}\\ +w_{4}\times \frac{\sum_{i\in A_{high}}\sum_{j=1}^{d}\left|{\left(B_{f_{1}}^{a}\right)}_{i,j}-{\left({\bar{B}}_{f_{1}}^{r}\right)}_{Y(A_{high}\left(i\right)),j}\right|}{n\times d} \end{array} \end{array}$$

where *A_high_* the set of scATAC‐seq cells connected by high‐weight anchors. *A_high_* (*i*) denotes the index of the scRNA‐seq cell paired with the *i*‐th scATAC‐seq cell through a high‐weight anchor. *Y*(*A_high_* (*i*)) denotes the cell cluster index of the scRNA‐seq cell indexed by *A_high_* (*i*). ${\bar{B}}_{f_{1}}^{r}$ denotes the matrix of cluster centroid embeddings of scRNA‐seq cells.

Similarly, SemiLT uses the loss function *L_low_* to enhance the similarity between cell–cell and cell–cluster pairs within low‐weight anchors.

(9) $$\begin{array}{c} \begin{array}{c} L_{low}\left(B_{f_{1}}^{a},B_{f_{1}}^{r}\right)=w_{5}\times (\frac{\sum_{i\in A_{low}}\sum_{j=1}^{d}\left|{\left(B_{f_{1}}^{a}\right)}_{i,j}-{\left(B_{f_{1}}^{r}\right)}_{A_{low}\left(i\right),j}\right|}{n\times d}\\ +w_{4}\times \frac{\sum_{i\in A_{low}}\sum_{j=1}^{d}\left|{\left(B_{f_{1}}^{a}\right)}_{i,j}-{\left({\bar{B}}_{f_{1}}^{r}\right)}_{Y(A_{low}\left(i\right)),j}\right|}{n\times d}) \end{array} \end{array}$$

Then, SemiLT also employs the loss function *L_batch_* to ensure the stability of batch correction.

(10) $$\begin{array}{ccc} L_{batch}\left(B_{f_{1}}^{a},B_{f_{1}}^{r}\right) & = & w_{6}\times \frac{\sum_{j=1}^{d}\left|\sum_{i=1}^{n}{\left(B_{f_{1}}^{a}\right)}_{i,j}-\sum_{i=1}^{n}{\left(B_{f_{1}}^{r}\right)}_{i,j}\right|}{n\times d}\\ & & +\frac{\sum_{j=1}^{d}\left|\sigma {\left(B_{f_{1}}^{a}\right)}_{\cdot ,j}-\sigma {\left(B_{f_{1}}^{r}\right)}_{\cdot ,j}\right|}{d} \end{array}$$

where *w*_4_,*w*_5_ and *w*_6_ are hyperparameters.

Finally, incorporate anchor information into *L_gas_*, making it perform a similar function as *L_rna_*:

(11)

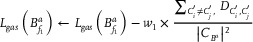

where $|C_{B^{a}}|$ denotes the number of distinct cell clusters to which scATAC‐seq cells in batch *B^a^* are anchored. $C_{i}^{\prime }$ denotes the set of scATAC‐seq cells anchored to cell cluster *C_i_*.

### Classification Loss

To learn discriminative features for various cell clusters, SemiLT employs the loss function *L_cf_* to supervise the learning of cell cluster in $B_{f_{2}}^{r}$.

(12) $$\begin{array}{ccc} L_{\mathrm{cf}}\left(B_{f_{1}}^{r},Y\right) & = & {\left(\frac{\sum_{i=1}^{n}{\left(B_{f_{2}}^{r}\right)}_{i,Y_{i}}}{n}\right)}^{-1}+\sum_{i\in B^{r}}\sum_{j=1}^{K}\left(1-I\left({\hat{y}}_{i}=y_{i}\right)\right)\\ & & \times I\left({\left(B_{f_{2}}^{r}\right)}_{i,j}>{\left(B_{f_{2}}^{r}\right)}_{i,Y_{i}}\right)\times {\left(B_{f_{2}}^{r}\right)}_{i,j} \end{array}$$

where *Y_i_* denotes the true cell cluster index of the *i*‐th scRNA‐seq cell, ${\hat{y}}_{i}$ denotes the predicted label for cell *i*, and *I* is an indicator function.

The final loss function we minimize is:

(13) $$\begin{array}{c} \begin{array}{c} L=L_{rna}+L_{gas}+L_{high}+L_{low}+L_{batch}+L_{cf} \end{array} \end{array}$$

### Label Transfer

The output of the neural network is a joint embedding space that aligns *X^r^* and *X^a^*. Then, a KNN classifier is used to compute probability scores for predicting the cell clusters of scATAC‐seq cells. The KNN score for assigning cell *i* to cell cluster *C_K_* is:

(14) $$\begin{array}{c} \begin{array}{c} {K_{score}}_{i,C_{K}}=\frac{\sum_{j\in M\left(i\right)}I\left(Y_{j}=C_{K}\right)}{\left|M\left(i\right)\right|},M\left(i\right)\in X^{r} \end{array} \end{array}$$

where *M*(*i*) represents the set of neighboring cells in *X^r^* for cell *i* in *X^a^*, and |*M*(*i*)| corresponds to the number of nearest neighbors.

SemiLT balance the KNN classifier by calculating the Euclidean distance from each cell in *X^a^* to each cell cluster centroid in *X^r^*. The Euclidean distance score for assigning cell *i* to cell cluster *C_K_* is:

(15) $$\begin{array}{c} \begin{array}{c} {E_{score}}_{i,C_{K}}=e^{-\left|d_{i,C_{K}}\right|} \end{array} \end{array}$$

where $d_{i,C_{K}}$ denotes the euclidean distance from cell *i* in *X^a^* to the centroid of cell cluster *C_K_* in *X^r^* in the embedding space.

The probability score for cell *i* being predicted as cell cluster *C_K_* is:

(16) $$\begin{array}{c} \begin{array}{c} P_{i,C_{K}}=s_{1}\times {K_{score}}_{i,C_{K}}+s_{2}\times {E_{score}}_{i,C_{K}} \end{array} \end{array}$$

where *s*_1_ and *s*_2_ denote the weights of two scores.

### Training Details

The batch size was set to 256 in all cases. Additional training details, including learning rate and number of training epochs used in each dataset, can be found in Note S3 (Supporting Information). The weights *s*_1_ and *s*_2_ were fixed at 0.2 and 0.8, respectively, across all datasets.

The weight *w*_1_ was set to $\mathrm{round}\left(\frac{K}{10}\right)\times \left(\mathrm{rare}+0.01\right)$, where round represents rounding (e.g., to the nearest integer), and rare indicates the proportion of rare cells in a batch size in scRNA‐seq. The weights *w*_2_ and *w*_3_ were set to $\mathrm{round}(\frac{K}{10})$ and $\frac{2}{K}$, respectively. The weights *w*_4_,*w*_5_ and *w*_6_ were fixed at 1.5, 0.8, 0.01, respectively, across all datasets.

### Evaluation Metrics

ARI:

ARI assesses the effectiveness of clustering by calculating the number of sample pairs assigned to the same or different clusters in both the true labels and the clustering results. The ARI score can be calculated as:

(17) $$ARI=\frac{RI-E\left(RI\right)}{\max\left(RI\right)-E\left(RI\right)}$$

where *E*(.) is the expectation, *RI* is the unadjusted rand index, which is defined as:

(18) $$RI=\frac{J_{a}+J_{b}}{C_{n}^{2}}$$

where *J_a_* is the number of cells that are assigned to the same cell cluster as benchmark labels, and *J_b_* is the number of cells that are assigned to different cell clusters as benchmark labels.

Recall:

Recall is the proportion of actual positive instances that were correctly predicted by the model. The Recall can be calculated as:

(19) $$\mathrm{Recall}=\frac{\mathrm{TP}}{\mathrm{TP}+\mathrm{FN}}$$

where TP (true positives) denotes the number of instances that are truly positive and are correctly predicted as such by the model. FN (false negatives) denotes the number of instances that are actually positive but are incorrectly predicted as negative. In this context, "positive" refers to instances that truly belong to a given class or label, and "negative" refers to instances that do not.

Precision:

Precision is the proportion of instances predicted as positive by the model that are positive. The Precision can be calculated as:

(20) $$\mathrm{Precision}=\frac{\mathrm{TP}}{\mathrm{TP}+\mathrm{FP}}$$

where FP (false positives) denotes the number of instances that are actually negative but are incorrectly predicted as positive. Here again, "positive" refers to instances that the model assigns to a particular class, while "negative" refers to those it does not.

F1 score:

The F1 score is the harmonic mean of precision and recall, providing a balanced measure of a model's performance. The F1 score can be calculated as:

(21) $$F1=2\times \frac{\mathrm{Precision}\times \mathrm{Recall}}{\mathrm{Precision}+\mathrm{Recall}}$$

AMI:

AMI stands for "Adjusted Mutual Information." It is a metric used to measure the similarity between two data clusterings by accounting for the chance or randomness in the clustering process. The AMI can be calculated as:

(22) $$\mathrm{AMI}\left(U,V\right)=\frac{\mathrm{MI}\left(U,V\right)-E\left[\mathrm{MI}\left(U,V\right)\right]}{\max\left(H\left(U\right),H\left(V\right)\right)-E\left[\mathrm{MI}\left(U,V\right)\right]}$$

where MI(*U*, *V*) is the mutual information between clusters *U* and *V*. *E*[MI(*U*, *V*)] is the expected mutual information between *U* and *V* under a random clustering assumption. *H*(*U*) and *H*(*V*) are the entropies of the clusterings *U* and *V*, respectively.

### Silhouette Coefficient

The Silhouette Coefficient is a metric used to assess the quality of clustering. It combines both cohesion and separation. The Silhouette coefficient can be calculated as:

(23) $$S\left(i\right)=\frac{b\left(i\right)-a\left(i\right)}{\max\left(a\left(i\right),b\left(i\right)\right)}$$

where *a*(*i*) is the average distance between the point *i* and all other points within the same cluster. *b*(*i*) is the minimum average distance from the point *i* to all points in any other cluster.

More detailed explanations on the model settings can be found in Note S4 (Supporting Information).

## Conflict of Interest

The authors declare no conflict of interest.

## Author Contributions

Z.C. and M.D. contributed equally to this work and are co‐first authors. Contributions: I) conception and design: Z.C. and B.L. II) Data collection and programming: Z.C. and M.D. III) Manuscript writing: Z.C., X.W., and B. IV) Final approval of manuscript: All authors.

## Code Availability

The source code in this paper can be found at https://github.com/Gut2Sdu/SemiLT‐release.

## Supporting information



Supporting Information

## Data Availability

All single‐cell datasets used in this paper are publicly available. Data‐1,2,3,4 was downloaded from (https://www.ncbi.nlm.nih.gov/geo/query/acc.cgi?acc=GSE194122). Data‐5 was downloaded from (https://github.com/caokai1073/uniPort). Data‐6: The scRNA‐seq dataset was downloaded from (https://tabula‐muris.ds.czbiohub.org/), the sci‐ATAC‐seq dataset was downloaded from (https://atlas.gs.washington.edu/mouse‐atac/). Data‐7 was downloaded from (https://github.com/SydneyBioX/scJoint). Data‐8: The scRNA‐seq dataset was downloaded from (https://github.com/dpeerlab/Palantir/), the sc‐ATAC‐seq dataset was downloaded from (https://gitlab.com/cvejic‐group/integrative‐scrna‐scatac‐human‐foetal). Data‐9 was downloaded from (https://satijalab.org/seurat/archive/v3.0/atacseq_integration_vignette.html).
