# Supplementary material for: SemiLT: A Multianchor Transfer Learning Method for Cross‐Modality Cell Label Annotation from scRNA‐seq to scATAC‐seq
Source: Adv Sci (Weinh). 2025 Sep 2;12(43):e07846. doi: 10.1002/advs.202507846 (PMC12631852; doi:10.1002/advs.202507846)
Supplement: Supplementary file 1 — Supporting Information [file ADVS-12-e07846-s001.docx]

*Supplementary Information*

**SemiLT: a multi-anchor transfer learning method for cross-modality cell label annotation from scRNA-seq to scATAC-seq**

# Supplementary Figures


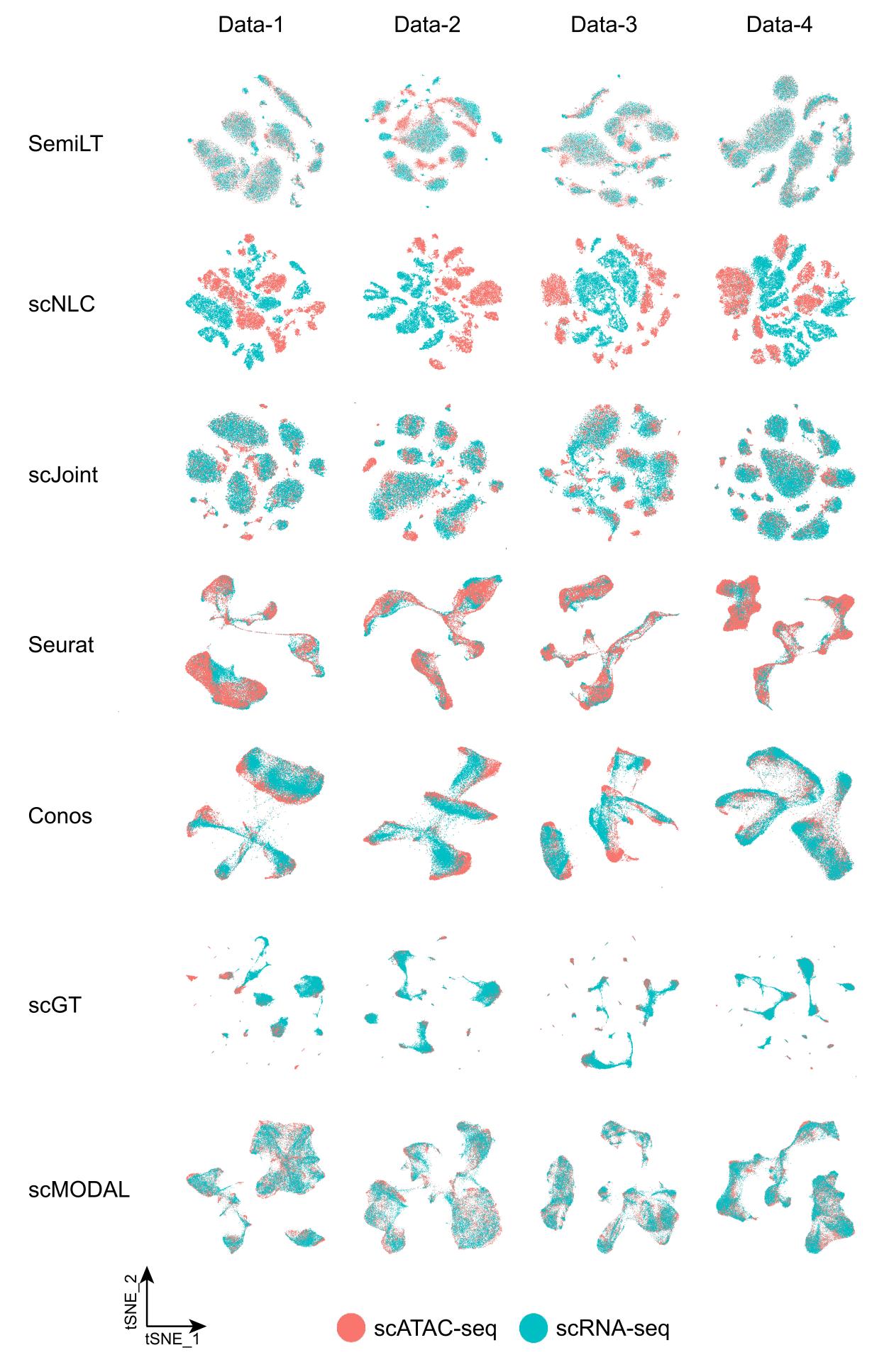


**Supplementary Fig S1.** tSNE visualization of SemiLT, scNLC, scJoint, Seurat, Conos, scGT and scMODAL integrated Data-1,2,3,4 generated from scRNA-seq and scATAC-seq coloured by technology (NeuCA did not output low-dimensional embeddings).

.


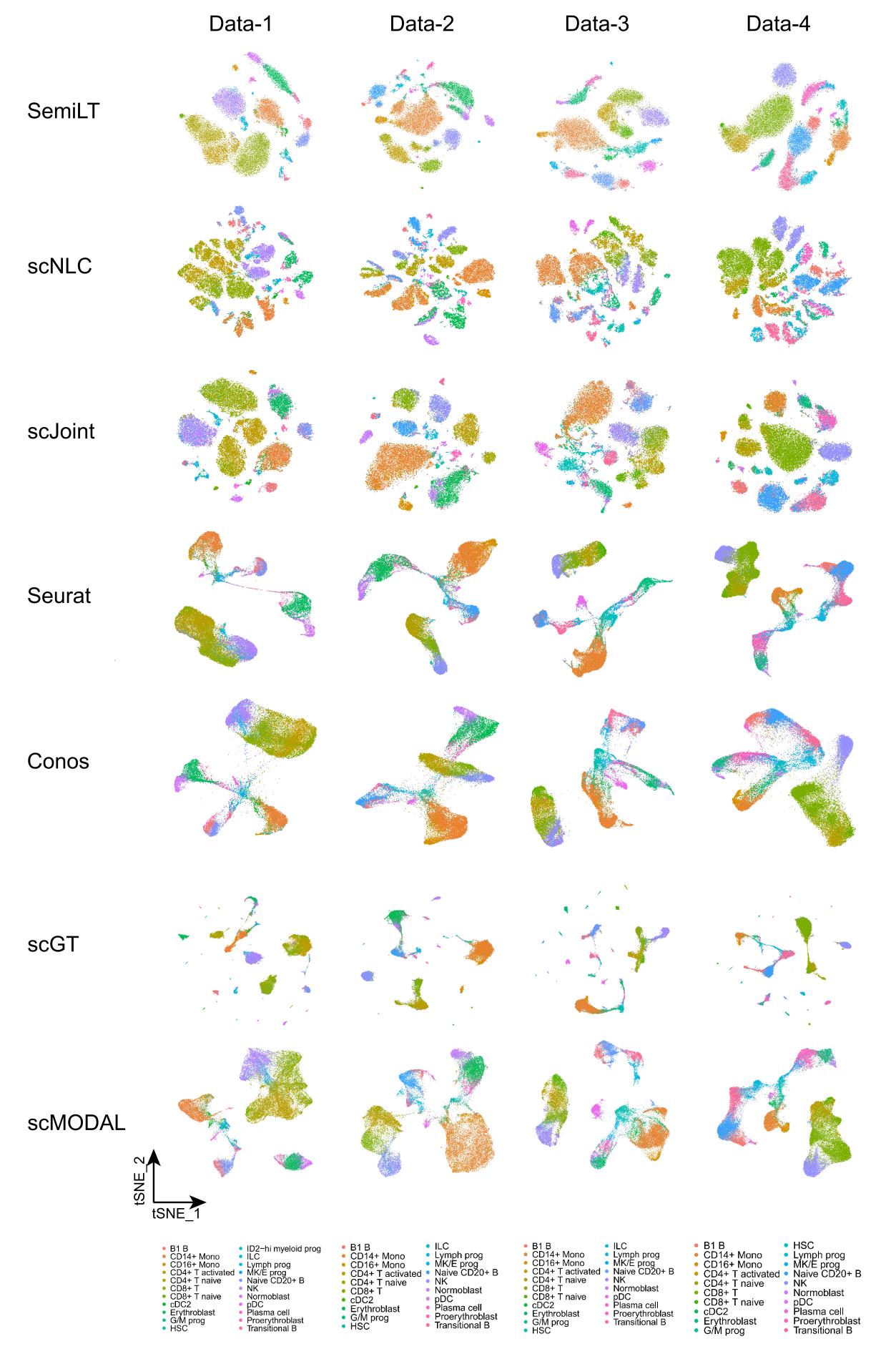


**Supplementary Fig S2.** tSNE visualization of SemiLT, scNLC, scJoint, Seurat, Conos, scGT and scMODAL integrated Data-1,2,3,4 generated from scRNA-seq and scATAC-seq colored by true cell labels (NeuCA did not produce low-dimensional embeddings).


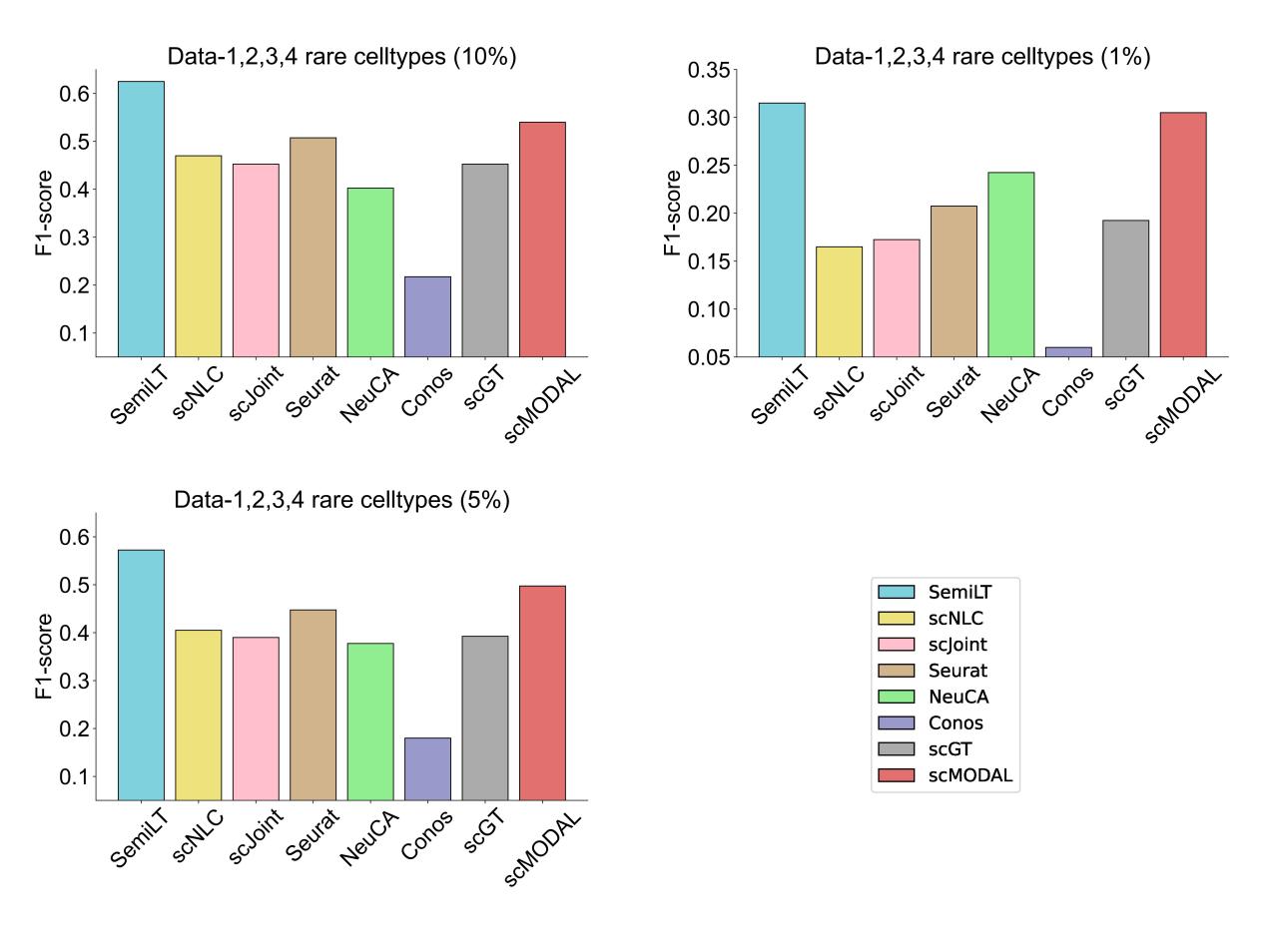
**Supplementary Fig S3.** F1 score for the prediction of rare cell types (10%, 5%, 1%) across Data-1,2,3,4.


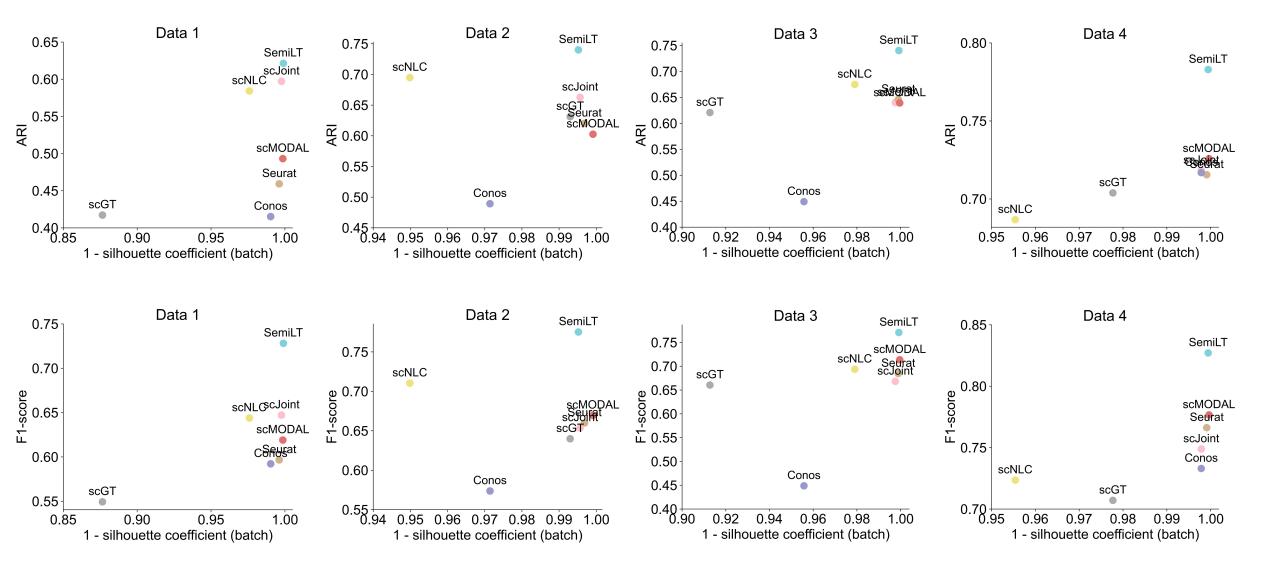


**Supplementary Fig S4.** ARI, F1-score and modality silhouette coefficients of SemiLT and other methods across Data-1,2,3,4.


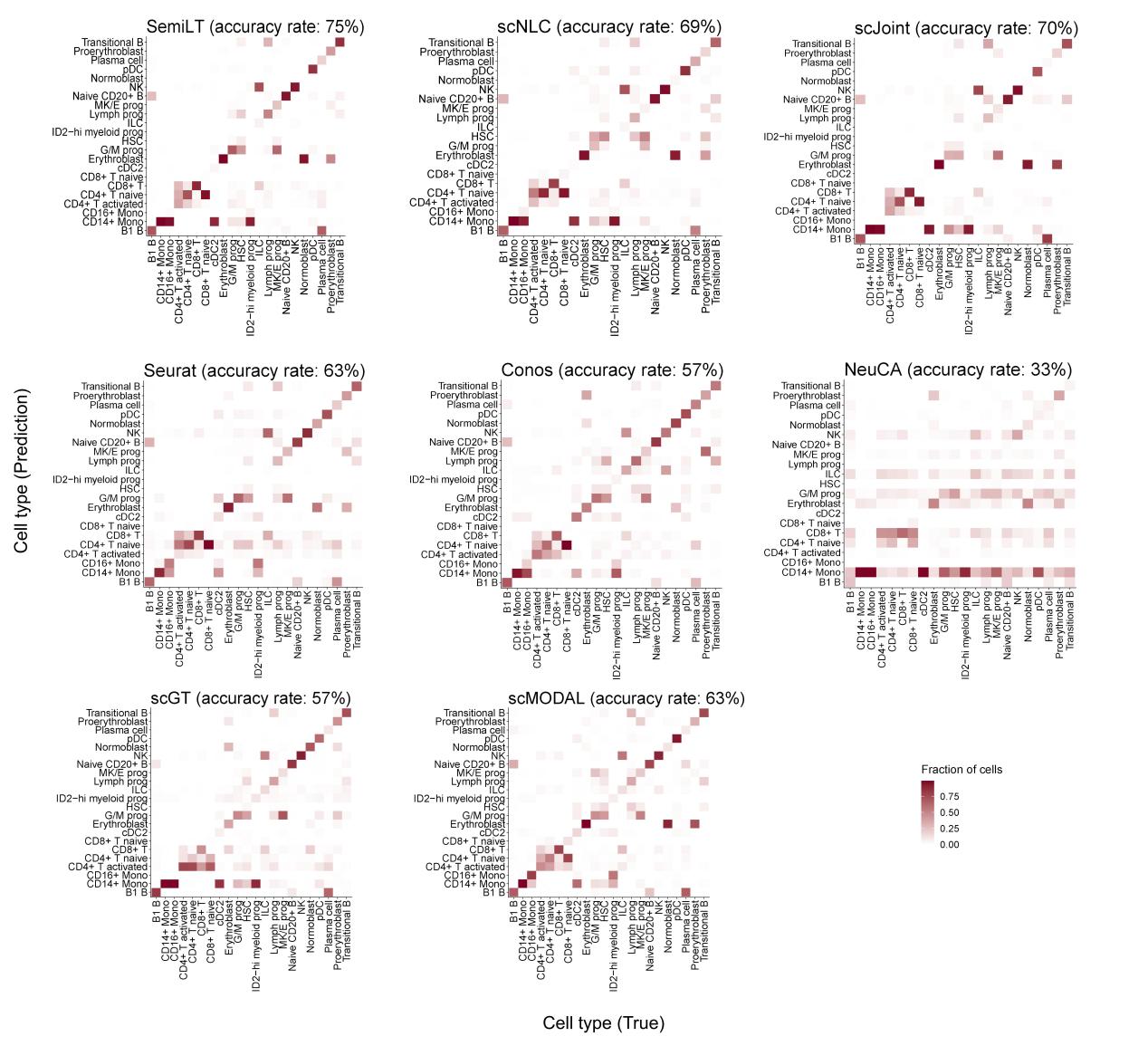


**Supplementary Fig S5.** Heatmaps comparing the true cell labels and the transferred cell labels of SemiLT, scNLC, scJoint, Seurat, Conos, NeuCA, scGT, and scMODAL in Data-1. A clearer diagonal structure indicates better agreement (row normalized). Cell types are sorted bottom-up according to their index in the scRNA-seq.


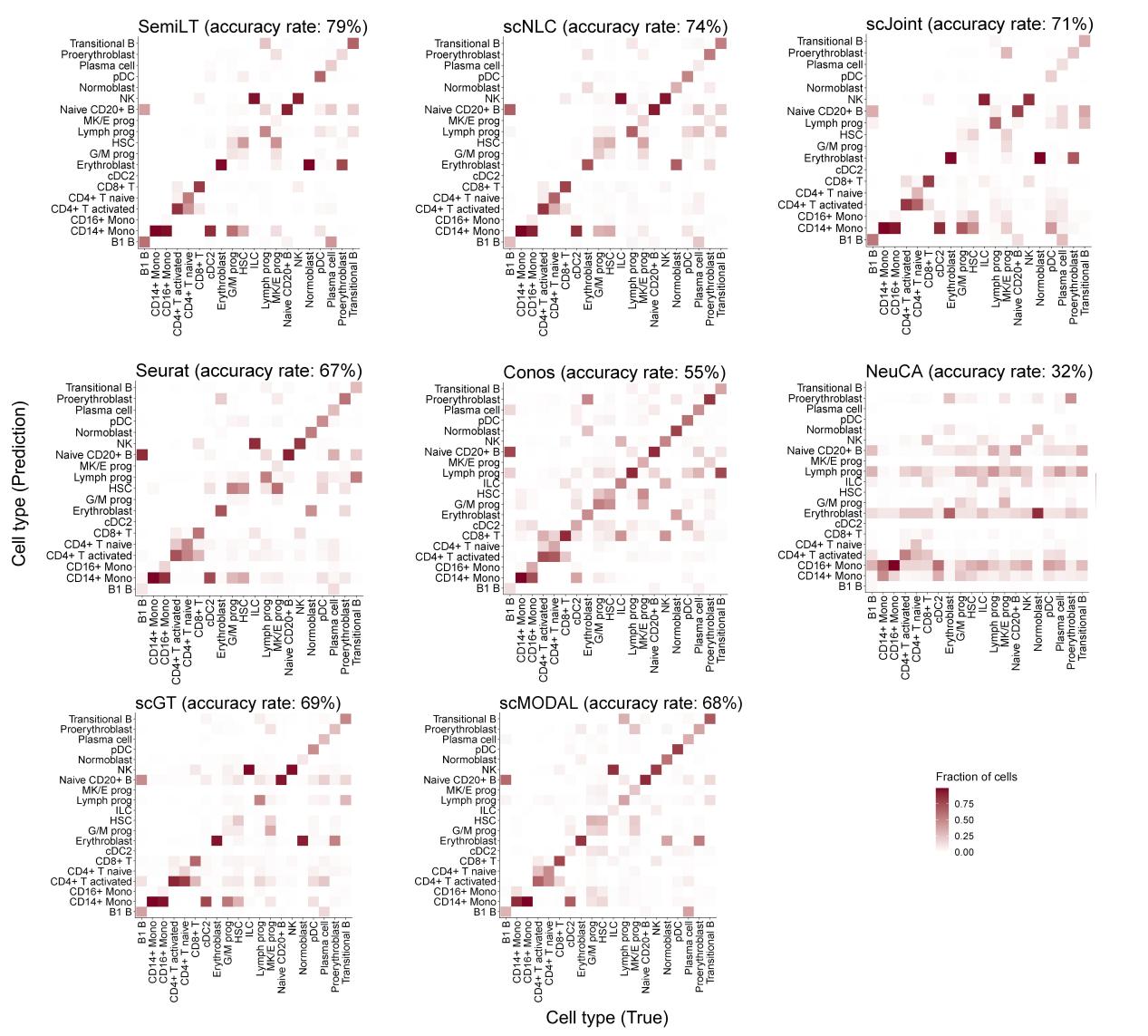


**Supplementary Fig S6.** Heatmaps comparing the true cell labels and the transferred cell labels of SemiLT, scNLC, scJoint, Seurat, Conos, NeuCA, scGT, and scMODAL in Data-2. A clearer diagonal structure indicates better agreement (row normalized). Cell types are sorted bottom-up according to their index in the scRNA-seq.


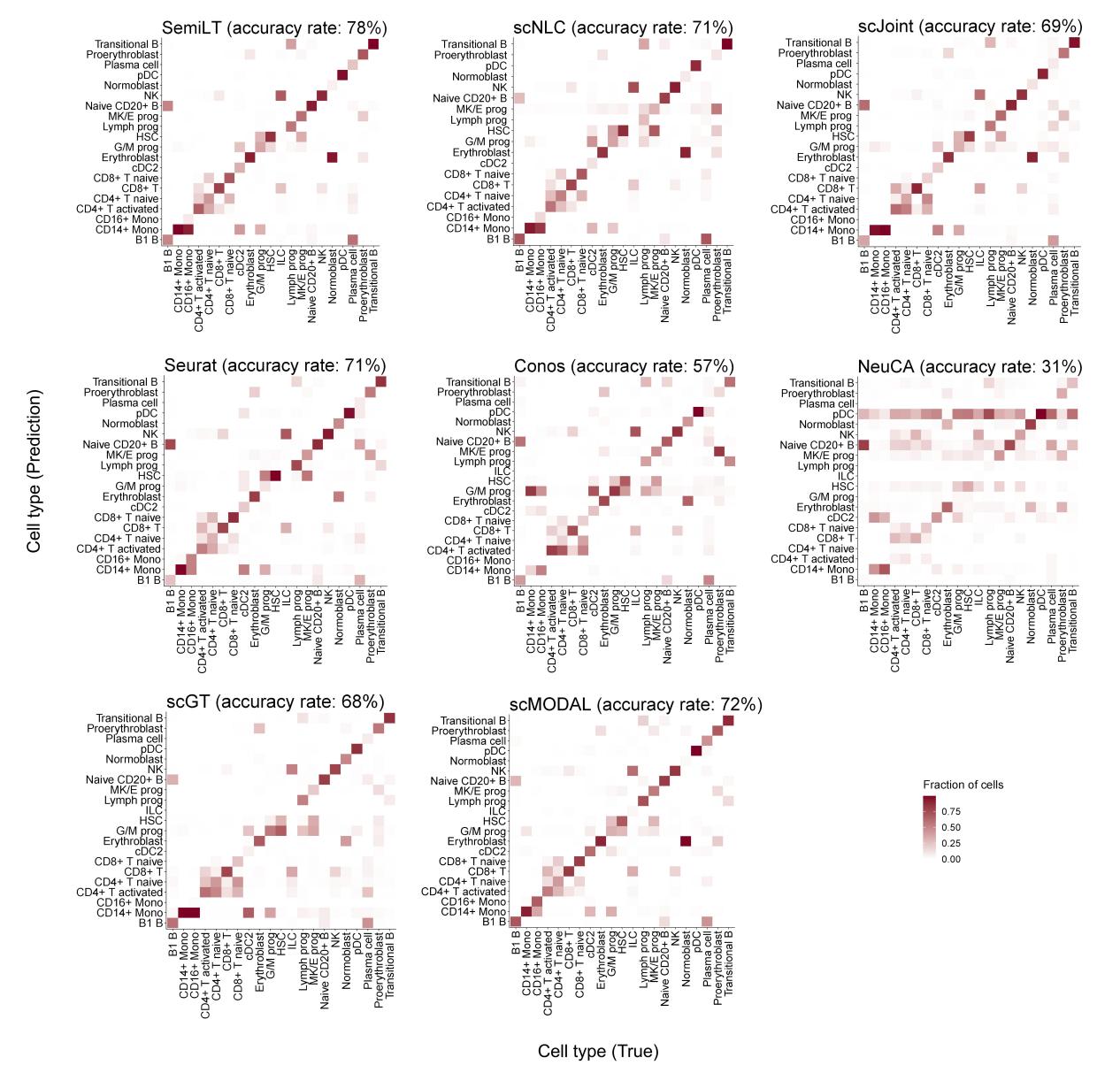


**Supplementary Fig S7.** Heatmaps comparing the true cell labels and the transferred cell labels of SemiLT, scNLC, scJoint, Seurat, Conos, NeuCA, scGT, and scMODAL in Data-3. A clearer diagonal structure indicates better agreement (row normalized). Cell types are sorted bottom-up according to their index in the scRNA-seq.


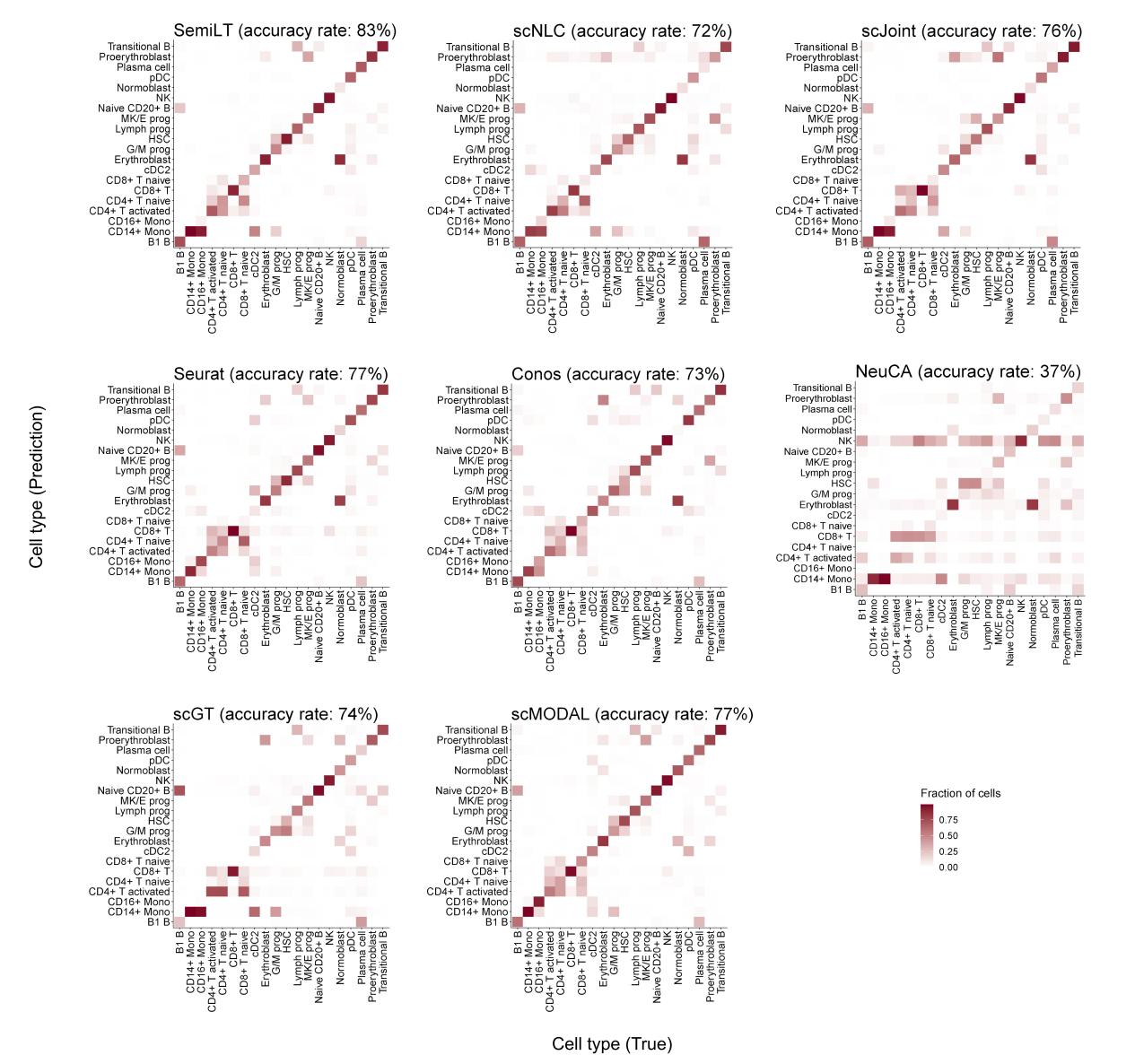


**Supplementary Fig S8.** Heatmaps comparing the true cell labels and the transferred cell labels of SemiLT, scNLC, scJoint, Seurat, Conos, NeuCA, scGT, and scMODAL in Data-4. A clearer diagonal structure indicates better agreement (row normalized). Cell types are sorted bottom-up according to their index in the scRNA-seq.


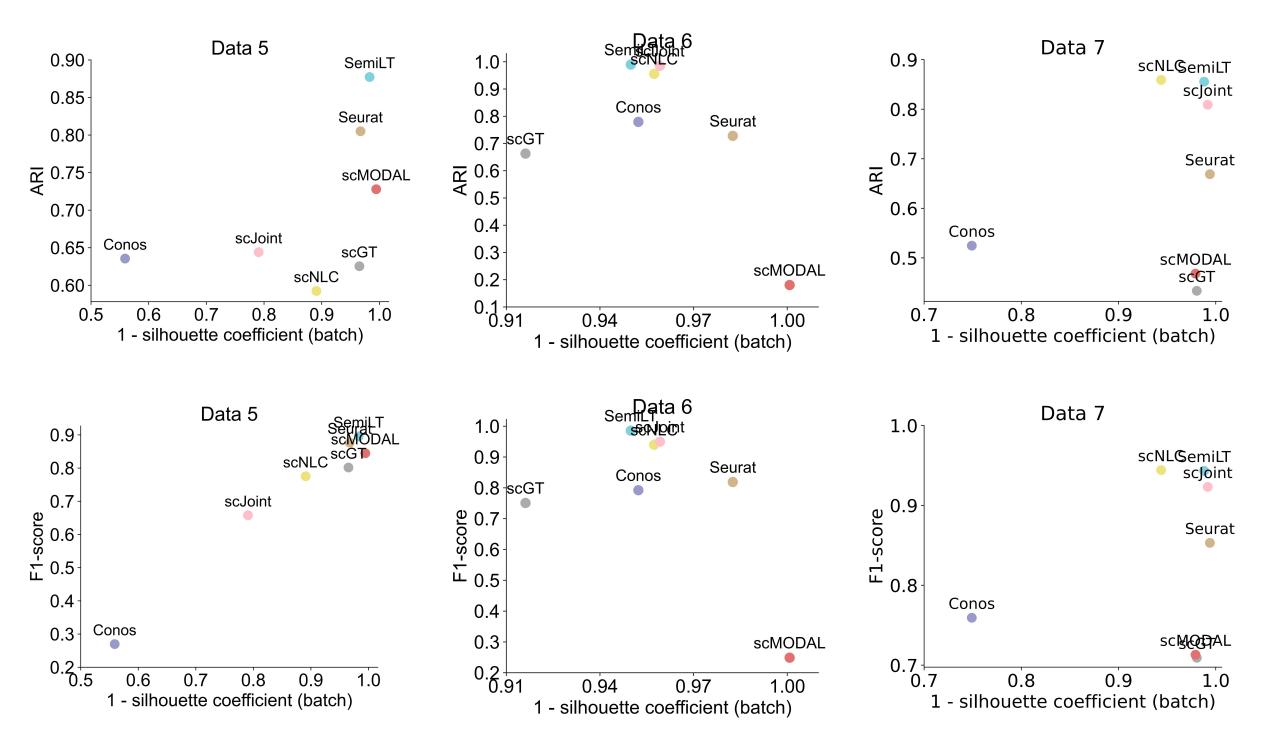


**Supplementary Fig S9.** AMI, F1-score and modality silhouette coefficients of SemiLT and other methods across Data-5,6,7.


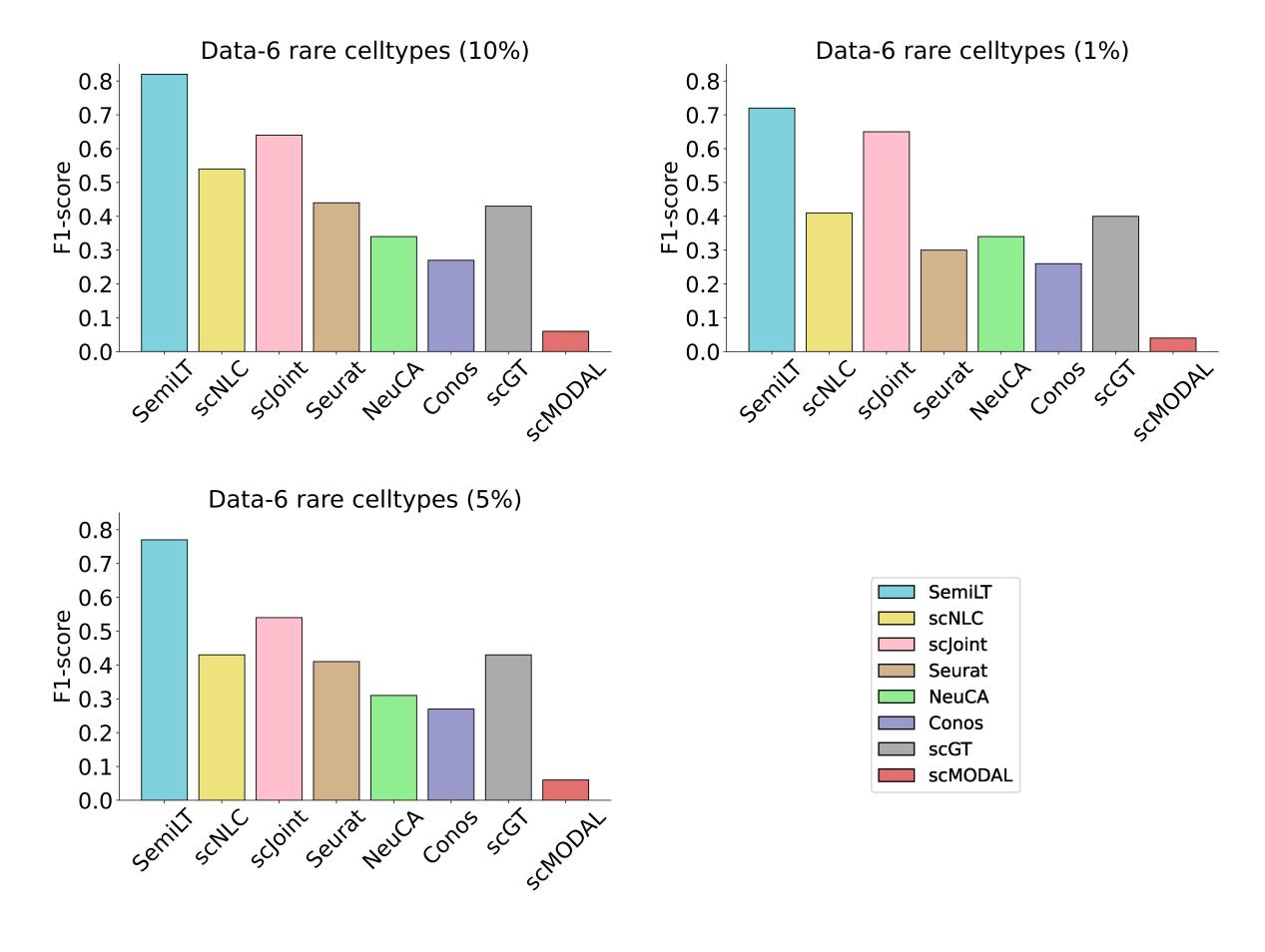


**Supplementary Fig S10.** F1 score for the prediction of rare cell types (10%, 5%, 1%) across Data-6.

**
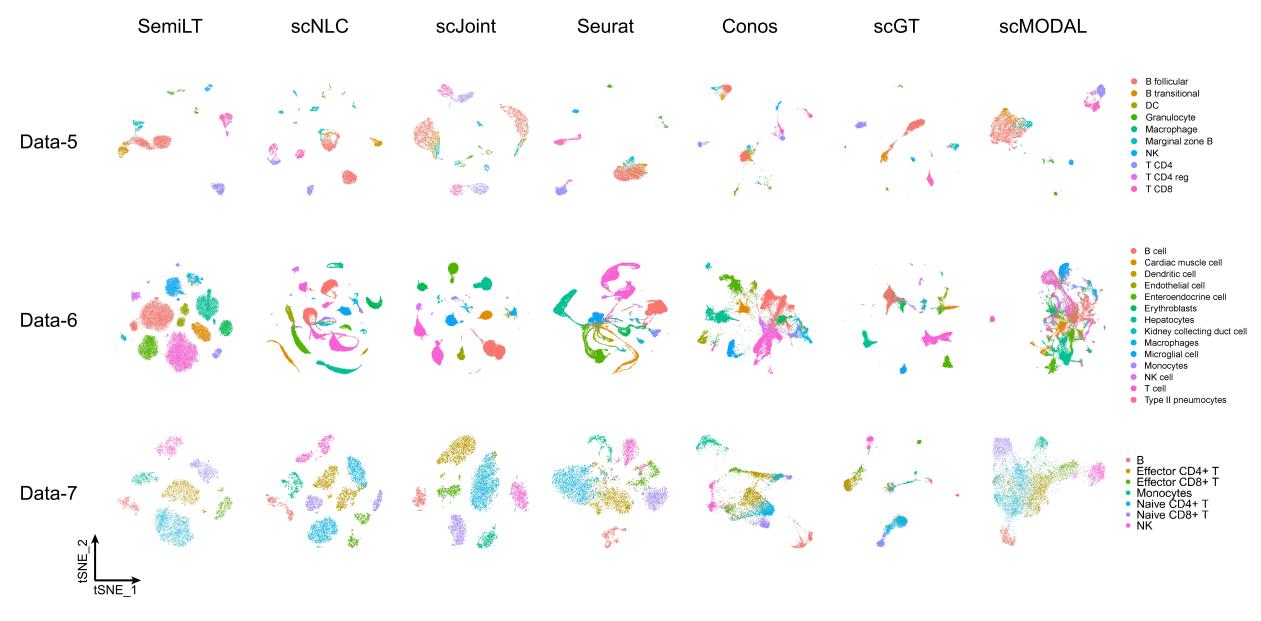
**

**Supplementary Fig S11.** tSNE visualization of SemiLT, scNLC, scJoint, Seurat, Conos, NeuCA, scGT, and scMODAL integrated Data-5,6,7 generated from scRNA-seq and scATAC-seq colored by true cell labels (NeuCA did not produce low-dimensional embeddings).


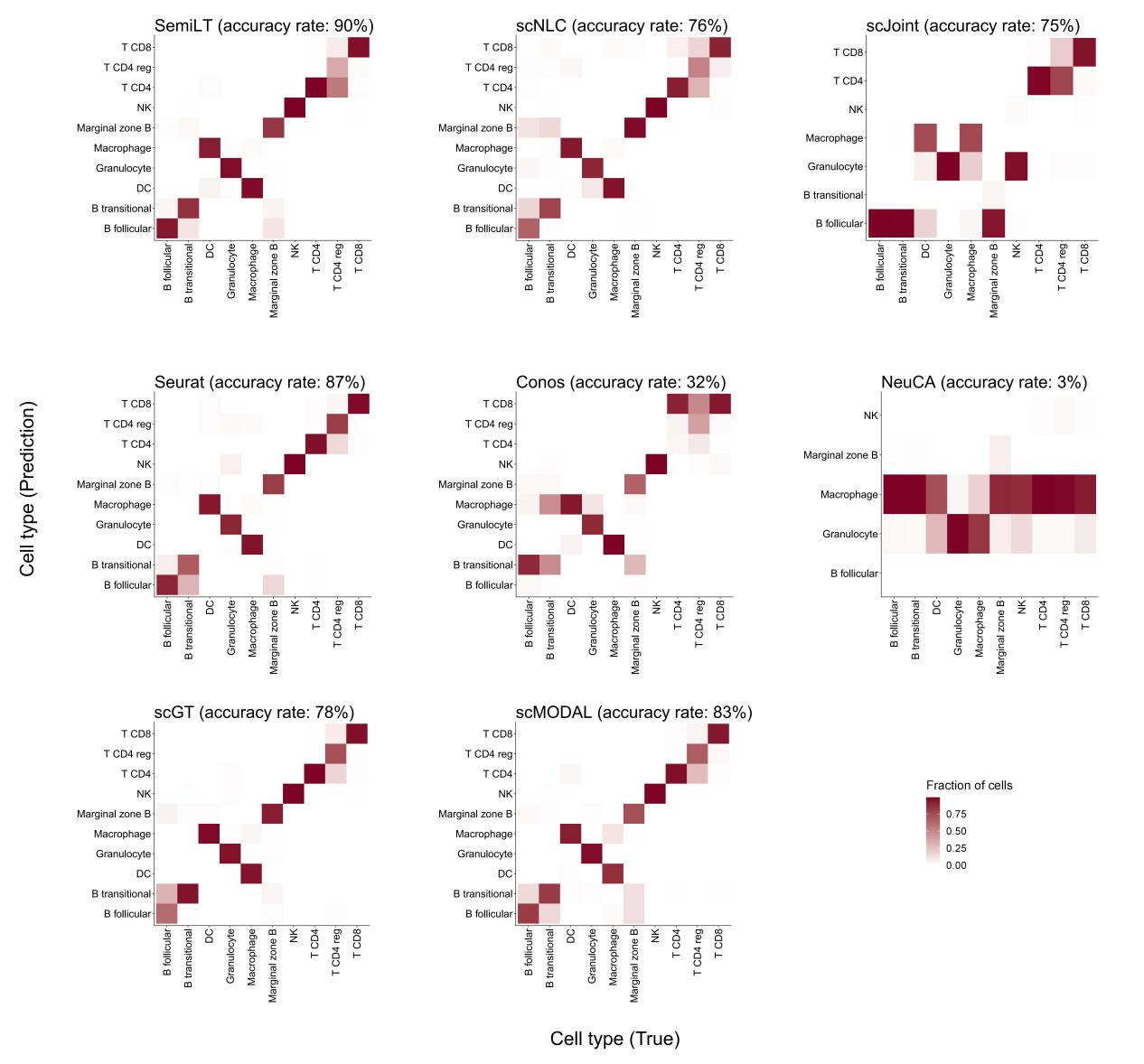


**Supplementary Fig S12.** Heatmaps comparing the true cell labels and the transferred cell labels of SemiLT, scNLC, scJoint, Seurat, Conos, NeuCA, scGT, and scMODAL in Data-5. A clearer diagonal structure indicates better agreement (row normalized). Cell types are sorted bottom-up according to their index in the scRNA-seq.


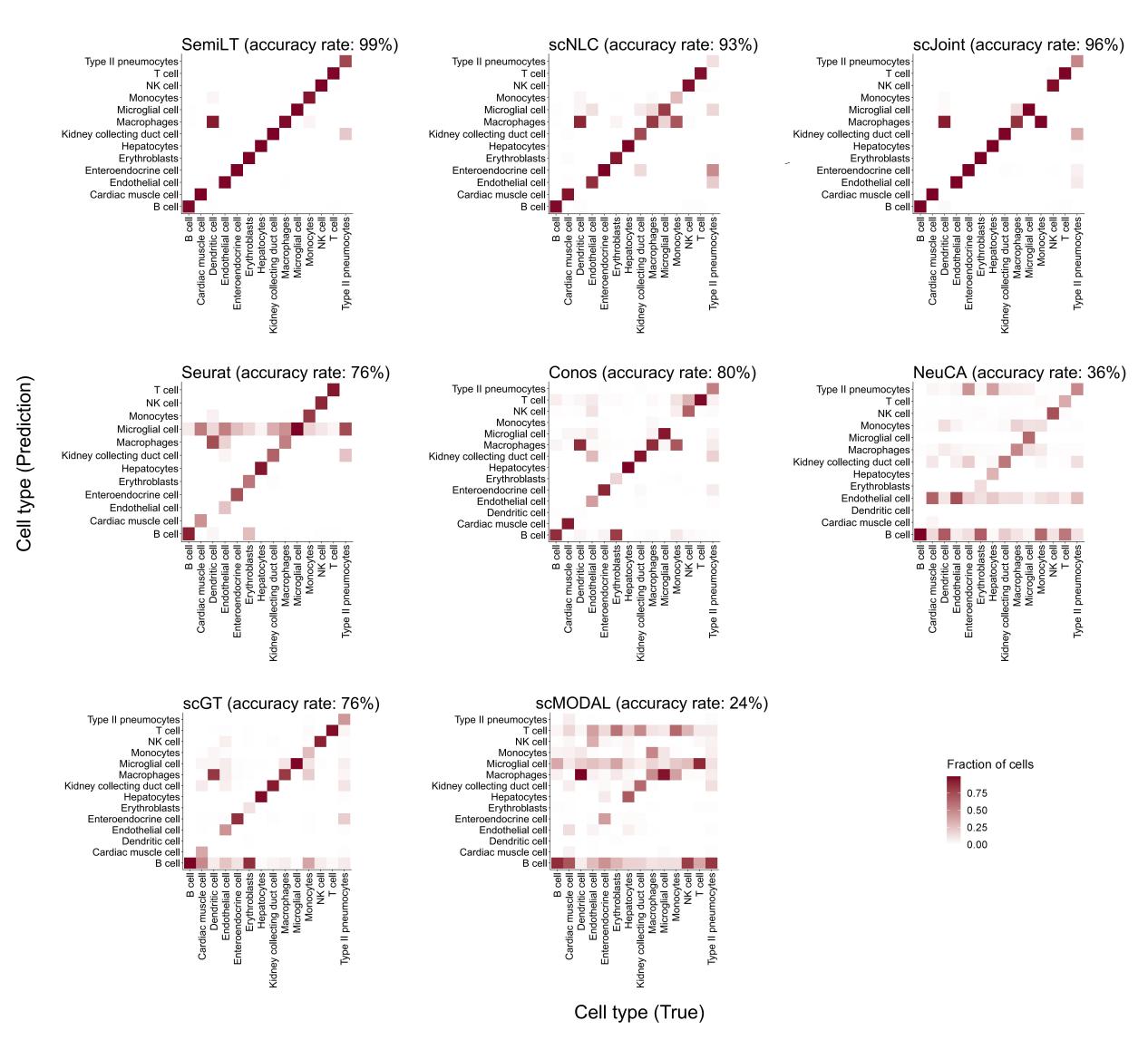


**Supplementary Fig S13.** Heatmaps comparing the true cell labels and the transferred cell labels of SemiLT, scNLC, scJoint, Seurat, Conos, NeuCA, scGT, and scMODAL in Data-6. A clearer diagonal structure indicates better agreement (row normalized). Cell types are sorted bottom-up according to their index in the scRNA-seq.


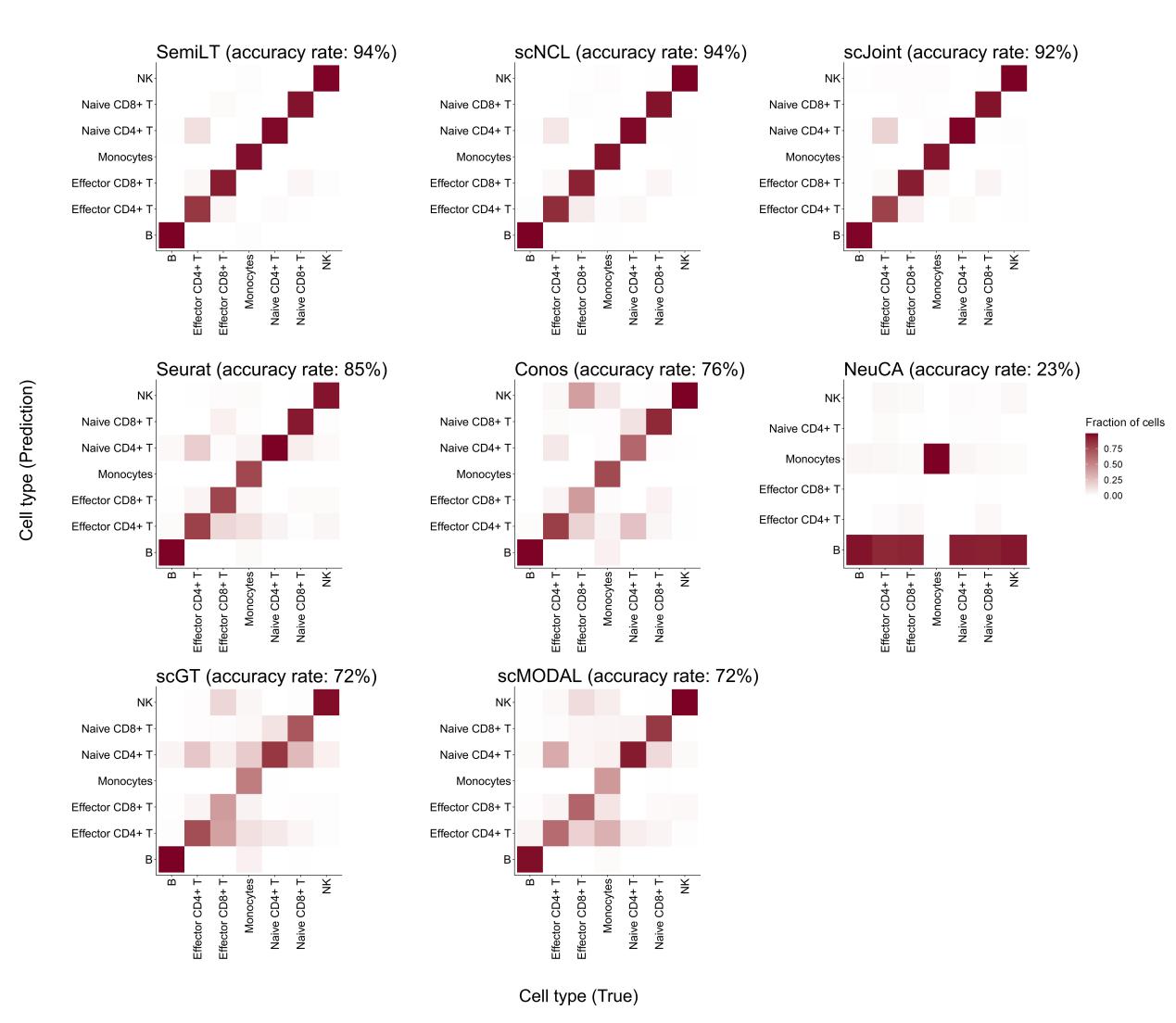


**Supplementary Fig S14.** Heatmaps comparing the true cell labels and the transferred cell labels of SemiLT, scNLC, scJoint, Seurat, Conos, NeuCA, scGT, and scMODAL in Data-7. A clearer diagonal structure indicates better agreement (row normalized). Cell types are sorted bottom-up according to their index in the scRNA-seq.


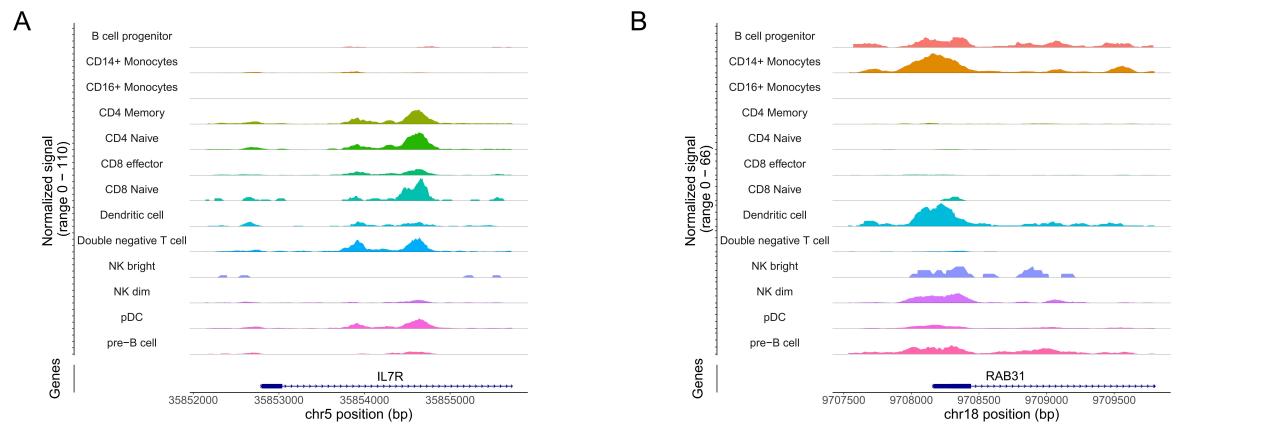
**Supplementary Fig S15.** Plot of Tn5 insertion frequency over the promoter region of IL7R and RAB31.

**
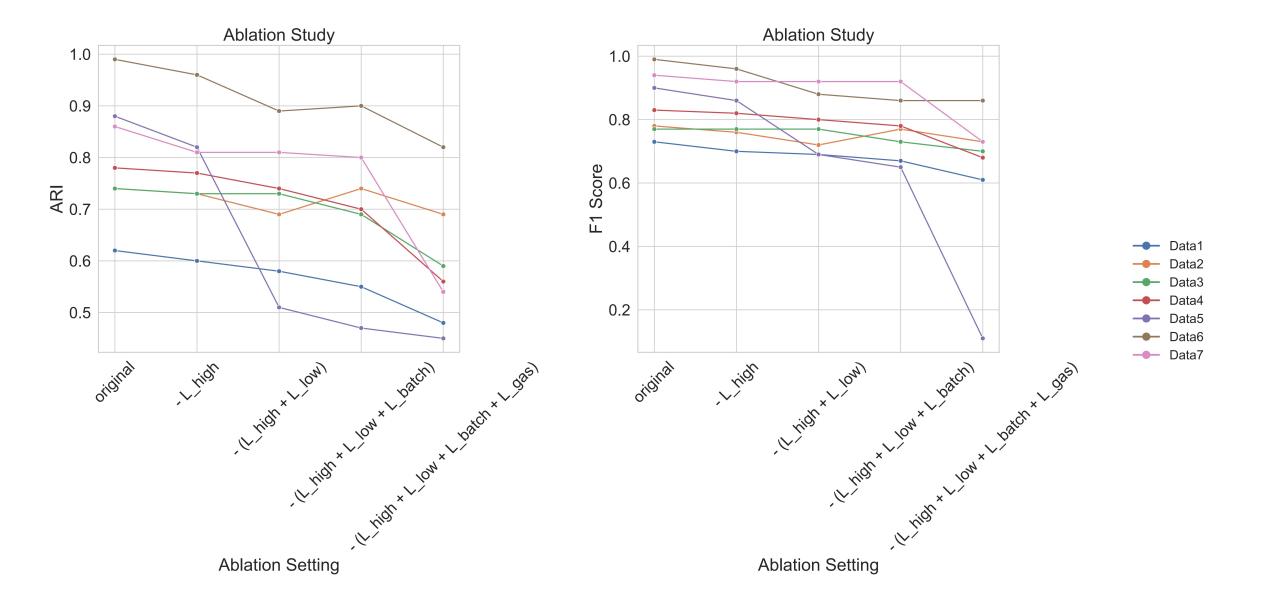
**

**Supplementary Fig S16.** The ablation study evaluated the impact of the loss function on the performance of cell type label transfer.


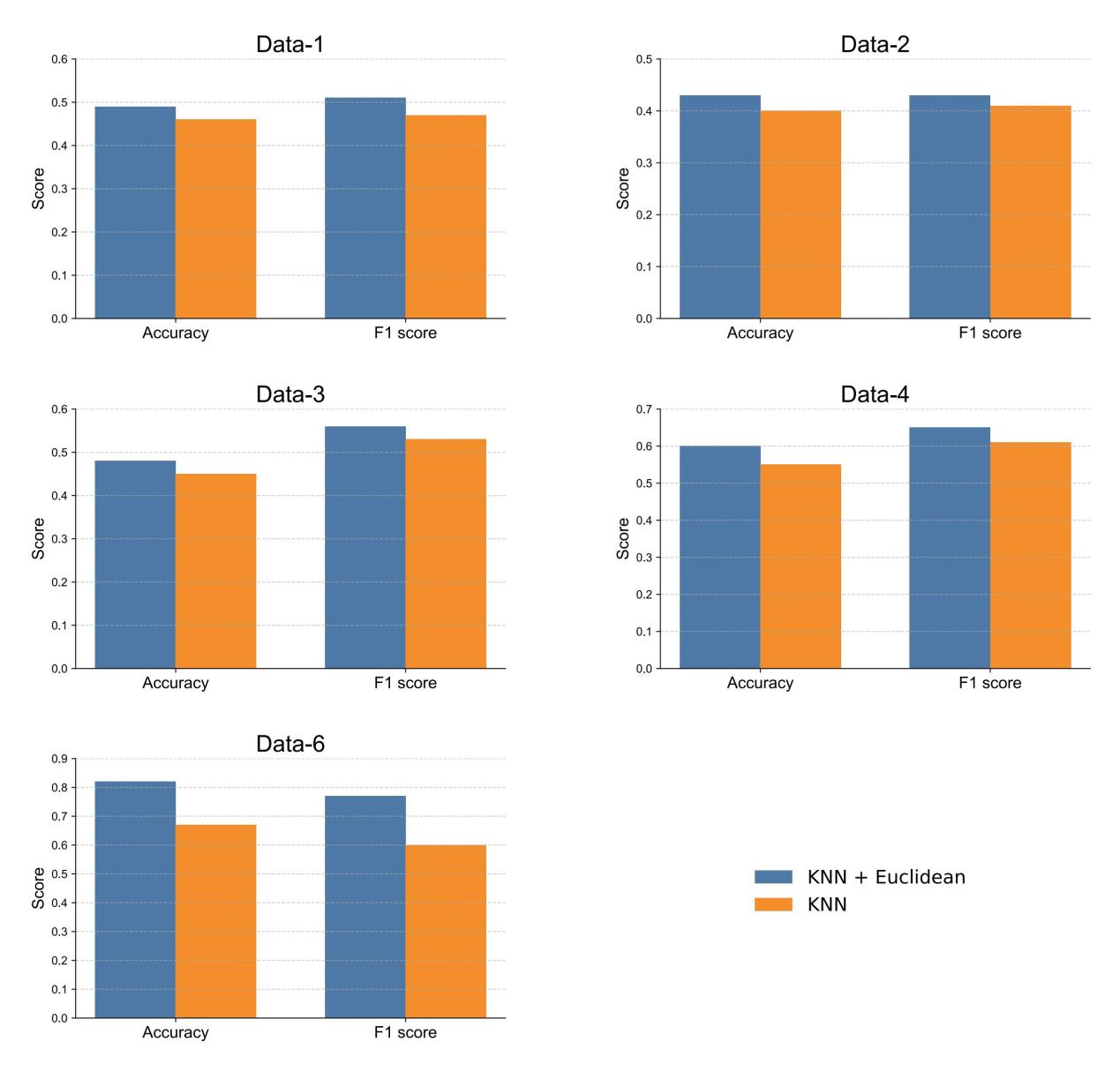


**Supplementary Fig S17.** The ablation study evaluated the impact of the Euclidean distance classifier on the prediction of rare cell types (3%).

# Supplementary Note

## N1 Parameter Combinations

For SemiLT:

- parameter 1 uses the default setting.
- parameter 2 modifies the *batch size* to 300.
- parameter 3 modifies the *learning rate* to 0.01.
- parameter 4 modifies *learning rate* to 0.01 and modifies *embedding_size* to 48.
- parameter 5 modifies the *batch size* to 200 and modifies the *learning rate* to 0.01.

For scNLC:

- parameter 1 uses the default setting.
- parameter 2 modifies the *KNN* to 5 and the *learning rate* to 0.005.
- parameter 3 modifies the data preprocessing method from *binarization* to *log normalization*.
- parameter 4 modifies *cont_w* to 0.1 and *cont_tau* to 0.3,
- parameter 5 modifies *cont_w* to 0.3 and *cont_tau* to 0.5.

For scJoint:

- parameter 1 uses the default setting.
- parameter 2 modifies $p$ to 0.9.
- parameter 3 modifies $p$ to 0.7.
- parameter 4 changes the *learning rate* to 0.005 and the *epoch* to 25.
- parameter 5 changes the *learning rate* to 0.008 and the *epoch* to 15.

For Seurat:

- parameter 1 uses the default setting.
- parameter 2 selects 3000 highly variable genes.
- parameter 3 selects 1500 highly variable genes.
- parameter 4 modifies *k.weight* to 30.
- parameter 5 modifies *k.weight* to 70.

For Conos:

- parameter 1 uses the default setting.
- parameter 2 modifies *KNN* to 30.
- parameter 3 modifies *KNN* to 15.
- parameter 4 modifies *KNN* to 30 and modifies $ncomps$ to 30.
- parameter 5 modifies *KNN* to 15 and modifies $ncomps$ to 30.

For NeuCA:

- parameter 1 uses the default setting.
- parameter 2 changes $model.size$ to $small$.
- parameter 3 changes $model.size$ to $big$.

For scGT:

- parameter 1 uses the default setting.
- parameter 2 modifies the *learning rate* to 3e-5.
- parameter 3 modifies the *learning rate* to 7e-5.
- parameter 4 modifies the *lamda1* to 0.2 and modifies the *lamda2* to 0.2.
- parameter 5 modifies the *hidden_channels* to 64 and modifies the *early_stop* to 40.

For scMODAL:

- parameter 1 uses the default setting.
- parameter 2 modifies the *Ir* to 0.0001.
- parameter 3 modifies the *Ir* to 0.01.
- parameter 4 modifies the *Ir* to 0.001 and modifies the *KNN* to 10.
- parameter 5 modifies the *Ir* to 0.001 and modifies the *KNN* to 3.

## N2 Data Preprocessing

- Data-1, 2, 3, 4 (human bone marrow mononuclear cells)

The gene expression, peak matrix and the cell-type annotation of the four paired scRNA-seq and scATAC-seq datasets were downloaded from <https://www.ncbi.nlm.nih.gov/geo/query/acc.cgi?acc=GSE194122>, where Data-1 contains 17,243 cells, Data-2 contains 15,226 cells, Data-3 contains 14,556 cells, and Data-4 contains 22,224 cells. The cell-type annotations were provided by the original paper.

- Data-5 (mouse spleen dataset)

The processed and annotated scRNA-seq data of mouse spleen was directly obtained from MultiMAP[1], containing 4,382 cells with 13,575 genes. The gene activity score of scATAC[2] was processed by SnapATAC[3], containing 3166 cells across 19,410 genes. We checked the cell-type annotations from both scRNA-seq and scATAC-seq studies manually and reannotated the labels such that the naming convention is consistent across the datasets. For example, the cell type ‘T CD4 naive’ in the scATAC-seq dataset was changed to ‘T CD4’. We also combined some of the cellular subtypes in the scATAC-seq data to increase the percentage of overlapping labels between two atlases for evaluation. More specifically, we combined ‘NK CD27+’ and ‘NK CD27-’ into ‘NK’ , and combined ‘T CD8 Memory’ and ‘T CD8 naive’ into ‘T CD8’.

- Data-6 (mouse cell atlas subset dataset)

The processed gene expression matrix and the cell-type annotation of the Tabula Muris mouse data of scRNA-seq were downloaded from <https://tabula-muris.ds.czbiohub.org/>. The quantitative gene activity score matrix and the cell-type annotation of the mouse sci-ATAC-seq atlas were downloaded from <https://atlas.gs.washington.edu/mouse-atac/>. We obtained the training data from the intersection of mouse organs covered by both datasets, including Kidney, Heart, Liver, Lung, Marrow, Spleen, Brain, Large Intestine, and Thymus. The scRNA-seq data contains 22,660 cells, and the sci-ATAC-seq data contains 36,770 cells.

- Data-7 (CITE-seq and ASAP-seq dataset)

The processed CITE-seq, ASAP-seq and the cell-type annotation were downloaded from <https://github.com/SydneyBioX/scJoint>, where the CITE-seq contains 4,644 cells and the ASAP-seq contains 4,502 cells.

- Data-8 (human developmental hematopoiesis dataset)

The scRNA-seq data and the cell-type annotation of the human developmental hematopoiesis dataset was downloaded from <https://github.com/dpeerlab/Palantir/>, which containing 24,327 cells. The scATAC-seq data, which lack ground-truth labels, were downloaded from <https://gitlab.com/cvejic-group/integrative-scrna-scatac-human-foetal>, which containing 3,611 cells.

- Data-9 (human peripheral blood mononuclear cell dataset)

The processed scRNA-seq, scATAC-seq and the cell-type annotation of scRNA-seq were downloaded from <https://satijalab.org/seurat/archive/v3.0/atacseq_integration_vignette.html>, where the scRNA-seq contains 9,432 cells and the scATAC-seq contains 8,728 cells.

For SemiLT, all gene expression matrices and gene activity score matrices were converted into binary form, with 1 indicating any nonzero original values. This binarization was applied as the final input for training. By binarizing, both modalities were scaled to have identical value ranges, which helps reduce data noise and facilitates easier cotraining. In addition, existing studies have shown that cell clustering based on binarized single-cell data has biological significance [4, 5].

For scATAC-seq data with accessible fragment files, we recommend using Signac [6] to calculate gene activity scores. In cases where fragment files are unavailable, we suggest using MAESTRO [7] to calculate gene activity scores as an alternative. Regarding the datasets used in this study:

1. Data-1, 2, 3, 4, 5, 6, and 7 provided scATAC-seq gene activity scores in their original studies.
2. Data-8, which lacked fragment files, had its gene activity scores computed using MAESTRO.
3. Data-9, which included fragment files, had its gene activity scores computed using Signac.

## N3 Model Parameters

SemiLT uses a fully connected neural network to transfer cell labels from scRNA-seq to scATAC-seq. The default parameter settings are:

| Parameter | Value |
| --- | --- |
| batch_size | 256 |
| lr | 0.008 |
| lr_decay_epoch | 20 |
| epochs | 20 |
| embedding_size | 64 |
| momentum | 0.9 |

Where *batch_size* is the number of training examples utilized in one forward/backward pass. *lr* is the initial learning rate, which controls how much the model is updated during training. *lr_decay_epoch* is the number of epochs after which the learning rate is decayed (reduced). *epochs* is the total number of times the entire training dataset is passed forward and backward through the model. *embedding_size* is the dimensionality of the embedding vector used to represent input features. *momentum* is the momentum factor that helps accelerate gradient descent in the relevant direction and dampens oscillations.

## N4 Evaluation Metrics

ARI

The ARI is calculated using the **adjusted_rand_score** function within the Python package **sklearn.metrics**.

Recall

The Recall is calculated using the **recall_score** function within the Python package **sklearn.metrics**, with the parameter set to '**weighted**' to accommodate multi-class problems.

Precision

The Precision is calculated using the **precision_score** function within the Python package **sklearn.metrics**, with the parameter set to '**weighted**' to accommodate multi-class problems.

F1 score

The F1 score is calculated using the **f1_score** function within the Python package **sklearn.metrics**, with the parameter set to '**weighted**' to accommodate multi-class problems.

AMI

The AMI is calculated using the **adjusted_mutual_info_score** function within the Python package **sklearn.metrics**, with the default parameter.

Silhouette coefficient

The Silhouette coefficient is calculated using the **silhouette_score** function within the Python package **sklearn.metrics**, with the default parameter.

Hypothesis testing

Hypothesis testing was performed using the **ranksums** function from the **scipy.stats**, with the default parameter.

# References

1. Jain MS, Polanski K, Conde CD, Chen X, Park J, Mamanova L, Knights A, Botting RA, Stephenson E, Haniffa M: **MultiMAP: dimensionality reduction and integration of multimodal data.** *Genome biology* 2021, **22:**1-26.

2. Chen X, Miragaia RJ, Natarajan KN, Teichmann SA: **A rapid and robust method for single cell chromatin accessibility profiling.** *Nature communications* 2018, **9:**5345.

3. Fang R, Preissl S, Li Y, Hou X, Lucero J, Wang X, Motamedi A, Shiau AK, Zhou X, Xie F: **Comprehensive analysis of single cell ATAC-seq data with SnapATAC.** *Nature communications* 2021, **12:**1337.

4. Jiang R, Sun T, Song D, Li JJ: **Zeros in scRNA-seq data: good or bad? How to embrace or tackle zeros in scRNA-seq data analysis?** *BioRxiv* 2020**:**2020.2012. 2028.424633.

5. Qiu P: **Embracing the dropouts in single-cell RNA-seq analysis.** *Nature communications* 2020, **11:**1169.

6. Stuart T, Srivastava A, Madad S, Lareau CA, Satija R: **Single-cell chromatin state analysis with Signac.** *Nature methods* 2021, **18:**1333-1341.

7. Wang C, Sun D, Huang X, Wan C, Li Z, Han Y, Qin Q, Fan J, Qiu X, Xie Y: **Integrative analyses of single-cell transcriptome and regulome using MAESTRO.** *Genome biology* 2020, **21:**1-28.
